# The role of social, economic, and medical marginalization in cancer clinical trial participation inequities: A systematic review

**DOI:** 10.1017/cts.2024.677

**Published:** 2024-12-20

**Authors:** Grace Ann Hanvey, Hannah Johnson, Gabriel Cartagena, Duane E. Dede, Janice L. Krieger, Kathryn M. Ross, Deidre B. Pereira

**Affiliations:** 1 University of Florida, Department of Clinical and Health Psychology, Gainesville, FL, USA; 2 Yale University, Department of Psychiatry, New Haven, CT, USA; 3 Mayo Clinic Comprehensive Cancer Center, Jacksonville, FL, USA

**Keywords:** Cancer, clinical trials, health inequities, representation, underserved populations

## Abstract

Extant literature reveals how patients of marginalized social identities, socioeconomic status (SES), and medical experiences – especially patients of color and older adults – are underrepresented in cancer clinical trials (CCTs). Emerging evidence increasingly indicates CCT underrepresentation among patients of lower SES or rural origin, sexual and gender minorities, and patients with comorbid disability. This review applies an intersectional perspective to characterizing CCT representativeness across race and ethnicity, age, sexual and gender identity, SES, and disability. Four databases were systematically queried for articles addressing CCT participation inequities across these marginalizing indicators, using the Preferred Reporting Items for Systematic Reviews and Meta-Analyses (PRISMA) guidelines. One hundred one articles were included in a qualitative evaluation of CCT representativeness within each target population in the context of their intersectional impacts on participation. Findings corroborate strong evidence of CCT underrepresentation among patients of color, older age, lower SES, rural origin, and comorbid disabling conditions while highlighting systemic limitations in data available to characterize representativeness. Results emphasize how observed inequities interactively manifest through the compounding effects of minoritized social identity, inequitable economic conditions, and marginalizing medical experiences. Recommendations are discussed to more accurately quantify CCT participation inequities across underserved cancer populations and understand their underpinning mechanisms.

## Introduction

Despite the necessity of representative cancer clinical trials (CCTs) to optimize equitable progress in cancer outcomes, overall CCT participation is remarkably low [1,2]. Strong evidence reveals that these low rates of CCT participation are still further compromised among individuals experiencing social, economic, and/or medical marginalization, particularly among patients of color [3–7] and older adults [1,8,9]. Further research increasingly suggests potential underrepresentation in CCTs among other marginalized populations, including sexual and gender minority (SGM) patients [10,11], patients of lower socioeconomic status (SES) [12,13], and patients experiencing greater disability in disease severity, comorbidity, or performance status [1,14,15]. However, systematic limitations in data collection and target variables addressed in prior literature render CCT inequities impacting these underserved groups more elusive [11,16–18], necessitating further research regarding the effects of these marginalizing characteristics on CCT participation. Considering the disproportionate cancer burden imparted upon groups enduring these forms of marginalization [9,10,15,16,19–23], representative CCTs that are generalizable to these populations are of the utmost importance for achieving equitable cancer care outcomes and associated progress across sociodemographic divides.

While prior investigations of CCT participation inequities have explored their effects on various underserved communities in cancer as previously described, these studies have primarily applied a singular perspective to marginalizing characteristics. Specifically, most existing CCT participation literature is limited by inadequate regard for the interactivity of overlapping forms of disadvantage, which serves a critical role in understanding CCT representativeness among the underserved. Intersectionality as a theoretical framework – in its focus on interlocking oppressive systems at the social-structural level and their manifestation in individual experiences [24,25] – is an apt scaffold through which these inequities may be interactively explained. However, despite increasing emphasis on the criticality of an intersectional approach to contextualizing public health outcomes [26], this framework has yet to be directly applied to CCT participation inequities.

This systematic review aims to provide a more comprehensive, ecologically valid characterization of CCT participation inequities to date across social, economic, and medical vectors of marginalization. In examining their independent and multiplicative influences through an intersectional lens, the authors seek to illustrate how race and ethnicity, age, sex, SGM status, SES, and diverse ability indicators have contributed to inequities in CCT participation across time.

## Materials and methods

### Search methods

This review adheres to Preferred Reporting Items for Systematic Reviews and Meta-Analyses (PRISMA) guidelines [27]. The first author developed and tailored a systematic search strategy to four databases, following general consultation with library sciences (Supplementary Table 1): (1) PubMed, (2) Web of Science, (3) PsycINFO, and (4) CINAHL. An initial search using this strategy was conducted on February 15, 2021, and then replicated on April 8, 2024, for newly published articles given significant time passage since the initial search. Covidence, a web-based collaboration software platform that streamlines the production of systematic and other literature reviews, was utilized to facilitate methodology [28].

### Eligibility criteria

Inclusion criteria for qualitative synthesis of results were (1) original research; (2) focus on CCT participation inequities regarding age, race and/or ethnicity, SES or one of its specific indicators (e.g., income, education, insurance, employment status), sexual identity, sex and/or gender identity, or ability status or relevant indicators (e.g., performance status, comorbidities); (3) peer-reviewed; and (4) full-text availability in English. Exclusion criteria required the removal of original protocols or reviews that (1) address trial participation disparities unrelated or nonspecific to individuals with cancer; (2) do not explicitly comment on CCT participation inequities; (3) encompass scope beyond the focus of this review, including papers exploring underlying barriers to identified inequities, developing solutions, and pediatric populations; (4) are case studies or reviews without quantitative analysis; or (5) are not peer-reviewed, full-text publications. Excluded papers per criterion four were scanned for eligible references unidentified by the search strategy.

### Data extraction procedures

Data extraction was standardized across three domains: (1) study characteristics, (2) methodology, and (3) sociodemographic reporting. The first domain specified the cancer population addressed, aims, sample size, intervention type(s), and target marginalizing indicators (Table 1). The second domain extracted information regarding study design, recruitment methods and databases, measures, and statistical procedures (Table 2). The third domain reported on available study information on sociodemographic characteristics relevant to the modes of marginalization addressed in this review (Table 3).

**Table 1.** Basic study characteristics

Authors	Year	Cancer site	Stage	Sample size	Aim of study	Trial type addressed	Primary target(s) of disparity	Study quality
Abbas et al.	2022	Gastrointestinal, hepato-pancreato-biliary	Any	10,518 CCT participants (36 CCTs) 2,255,730 CCT-eligible non-participants	Examine relationships between patient demographic and socioeconomic indicators, institutional factors, and CCT participation	Surgical	Age Race/ethnicity SES	5
Abi Jaoude et al.	2020	Any	Any	428,560 accruals (600 CCTs)	Evaluate (1) characteristics of phase III CCTs that use performance status exclusionary criteria, (2) use of such exclusionary criteria over time, and (3) trial representativeness by performance status.	Phase III, multi-arm, explicit supportive care inclusion	Ability and comorbidity	5
Acoba, Sumida, and Berenberg	2022	Any	Any	1,515 CCT participants 29,982 population controls	Examine CCT enrollment at a center prioritizing Asian and Native Hawaiian enrollment	Therapeutic, non-therapeutic	Race	5
Al Hadidi et al.	2022	Hematologic	Any	1057 total participants (7 CCTs)	Evaluate the representativeness of Black individuals with hematologic malignancies in CCTs for CAR-T therapies	CAR-T	Race	5
Aldrighetti et al.	2021	Breast Prostate Lung Colorectal	Any	5,867 participants (93 studies)	Assess the representativeness of breast, prostate, lung, and colorectal CCTs studying precision medicine	Precision oncology	Race/ethnicity	4
Ajewole et al.	2021	Lung, breast, prostate	Any	142 CCTs total 74 CCTs (total reporting race; 35,933 participants)	Evaluate reporting and inclusion of Black Americans in oral chemotherapy CCTs	Chemotherapeutic, oral	Race/ethnicity	5
Awad et al.	2020	Gynecologic (cervical, endometrial, multiple, ovarian)	Any	357 publications 9,492 patients 84 publications reporting race 2,483 patients	Describe the longitudinal representation of minority women in phase I, GYN oncology trials	Therapeutic, unspecified	Race	5
Baldini et al.	2022	Gastrointestinal, hematologic, thoracic	Any	1,319 total patients	Evaluate the representativeness of older adults (70+) in referrals to early-phase CCTs	Systemic, early-phase	Age	4
Baquet, Ellison, and Mishra	2009	Breast, colorectal, lung, lymphoma, leukemia, reproductive (sex-specific)	Any	2,240 CCT accruals	Identify relationships between sociodemographic characteristics and NCI-sponsored therapeutic CCT enrollment	Therapeutic, unspecified	Age Race Sex SES	5
Behrendt, Hurria, Tumyan, Niland, and Mortimer	2014	Breast	I–IV	1,482 total patients 446 accruals	Examine the degree to which SES and clinical factors confound disparities in CCT accrual	Adjuvant, neoadjuvant, non-adjuvant therapies	SES Race/ethnicity	5
Bero et al.	2021	Any (including sex-specific)	Any	122 CCTs total	Evaluate racial representativeness of radiation therapy CCTs	Radiation	Race/ethnicity Sex	4
Borad et al.	2020	Multiple myeloma	Any	50 (42 provided mean, 15 median)	Evaluate age representativeness of phase III, therapeutic multiple myeloma trials	Therapeutic, phase III	Age	4
Borno et al.	2019	Breast, colorectal, prostate	Any	3,580 CCT accruals 20,305 incident CCC cases 341,114 incident catchment area cases	Examine whether recruitment inequities were due to inadequate catchment area outreach or lack of representative cancer in the CCC Examine whether CCC data presentation obscures recruitment inequities among different cancer types	Therapeutic, unspecified	Age Race/ethnicity SES	5
Brierley et al.	2020	Myelodysplastic syndromes	Any	449 accruals 1,919 total patients	Evaluate baseline characteristics of patients with myelodysplastic syndromes accrued to CCTs	Therapeutic, unspecified	Age SES Sex Ability	5
Bruno et al.	2022	NSCLC, colorectal, breast	Advanced/metastatic	Total patients: NSCLC: 14,768 Colorectal: 7,879 Breast: 5,276	Evaluate racial representativeness of lung, breast, and colorectal CCTs and biomarker testing in the US	Any, unspecified	Race/Ethnicity	3
Bruno, Li, and Hess	2024	Lung	Advanced/ metastatic	3,845 total patients	Evaluate racial representativeness of CCTs and biomarker testing among individuals with metastatic lung cancer and Medicaid coverage	Any, unspecified	Race SES	4
Canoui-Poitrine et al.	2019	Colorectal	Any	577	Evaluate CCT availability, eligibility, invitation, enrollment, and associated reasons among older adults with colorectal cancer	Any therapeutic, diagnostic, or monitoring	Age ability and comorbidity	5
Casey et al.	2023	Lymphoma	Any/all	33 RCTs	Assess demographic and geographic representation of US lymphoma RCTs	Drug, therapeutic	Race/ethnicity Sex SES	5
Choradia et al.	2024	Any/all	Any/all	38,527 total patients	Evaluate inequities in demographic representativeness of NCI NIH Clinical Center CCTs	Therapeutic, unspecified	Race/ethnicity Age Sex	5
Costa, Hari, and Kumar	2016	Multiple myeloma	I–III	128 CCTs 8,869 accruals	Examine the representativeness of multiple myeloma CCTs in the USA	Therapeutic, any	Age Race/ethnicity Sex Ability	5
Craig, Gilbery, Herndon, Vogel, and Quinn	2010	Prostate	Any	211 accruals 37,216 patients	Assess the proportion of older adults with prostate cancer enrolled in Medicare who participated in CCTs Compare characteristics of non-CCT and CCT participants within the older adult Medicare prostate cancer population	Any, unspecified	Age Race/ethnicity Sex SES Ability	5
Diehl et al.	2011	Breast, thoracic, sarcoma	I–IV	10 trials	Examine sociodemographic accrual patterns across 10 surgical CCTs Compare characteristics of surgical CCTs successful and unsuccessful at recruiting minority patients	Surgical	Race/ethnicity	4
Dressler et al.	2015	Breast, Hodgkin’s lymphoma, gastric, colorectal, pancreatic, prostate	Any	7 CCTs 8,456 accruals	Examine patient- and institution-level factors contributing to participation in pharmacogenomic CCTs	Therapeutic, pharmacogenomic	Race/ethnicity	5
Du, Gadgeel, and Simon	2006	Lung	II–IV	91 accruals 427 total patients	Assess factors associated with CCT enrollment among lung cancer patients	Therapeutic, unspecified	Age Race/ethnicity Sex SES Ability	5
Dudipala et al.	2023	Any/all	Any	1121 total patients	Examine sociodemographic predictors of clinical trial discussion and enrollment among individuals with lung cancer at Boston Medical Center	Therapeutic, primary	Age Race/ethnicity	5
Duma et al.	2018	Breast, colorectal, lung, pancreas, prostate, renal, melanoma	Any	1,012 CCTs total 210 (CCTs total reporting race/ethnicity) 210 (CCTs total reporting race/ethnicity)	Evaluate sex-related, racial, and ethnic representativeness of oncology trials from 2003 to 2016.	Therapeutic, any oncology	Race/ethnicity Sex	5
Earl et al.	2023	Glioma	Any	570 CCT enrollees	Evaluate the impact of social determinants of health on CCT participation and their impact on geographical disparities	Therapeutic, biobanking	SES Geography	4
Elshami et al.	2022	Hepato-pancreato-biliary	I–IV	511,639 total patients	Evaluate sociodemographic and clinical predictors of CCT enrollment	Any	Race/ethnicity Age SES Ability and comorbidity	5
Eskander et al.	2022	Pancreatic	I–IV	1,127 enrollees 301,240 non-enrollees	Evaluate the impact of social determinants of health on CCT enrollment	Any	Age Race/ethnicity SES (and rurality) Ability and comorbidity	5
Fakhry et al.	2023	Any	Any	38 studies to review reporting 15 studies eligible for pt analysis (1,284 pts)	Evaluate racial and ethnic representativeness and associated reporting of phase 2, US proton therapy trials	Proton therapy	Race/ethnicity	5
Fayanju et al.	2019	Breast	Any	809,843 total patients 17,214 accruals 792,719 non-accruals	Compare cohort of current breast surgical oncology patients enrolled in CCTs and NCDB eligible patient non-accruals	Surgical	Race/ethnicity SES	5
Freudenburg et al.	2022	Bladder	Any	544 studies total 24 studies reporting race	Evaluate racial and ethnic representativeness of and associated reporting in bladder CCTs	Therapeutic, phase I–III	Race/ethnicity	5
Gopishetty, Kota, and Guddati	2020	Breast, colon, lung, DLBCL, AML, ALL	Any	103 race-reporting studies 69 age-reporting studies	Investigate age, race, and ethnicity distribution in phase III drug trials for the most common solid organ tumors and hematological cancers	Drug, therapeutic	Age Race/ethnicity	5
Gordis et al.	2022	HPV-associated OPSCC	Any	2,995 (32 trials) 14,805 comparison patients	Evaluate the representativeness of HPV-associated OPSCC trials relative to US national database	Any, unspecified	Age Race Sex Ability and Comorbidity	4
Grant et al.	2020	Breast, colorectal, lung, prostate	Any	168 trials (96 reporting on race/ethnicity) 34,329 accruals	Examine recent phase III US CCT enrollment inequities across race and ethnicity	Targeted systemic therapy, cytotoxic chemotherapy, radiation or surgery	Race/ethnicity	5
Green et al.	2022	Any/all	Any/all	8,360 CCT participants 420,983 non-participants	Evaluate the representativeness of older adults with cancer and Medicare FFS coverage in CCTs	Therapeutic, unspecified	Age SES Race/ethnicity Ability and comorbidity	5
Grette et al.	2021	Breast, gynecologic	Any	8,820 CCT participants (53 trials)	Evaluate racial representation in breast and gynecologic immunotherapy CCTs	Immunotherapy	Race	5
Gross, Filardo, Mayne, and Krumholz	2005	Breast	Any	737 accruals 7,384 non-accruals	Examine the impact of SES on CCT enrollment among older breast cancer patients	Drug, therapeutic	SES	4
Guerrero et al.	2018	Melanoma, breast, lung	Any	208 trials total (reporting race/ethnicity)	Evaluate racial and ethnic representativeness and associated reporting practices of various types of cancer research	Any, unspecified	Race/ethnicity	4
Hantel et al.	2022	Acute leukemia	Any	3,734 CCT enrollees	Evaluate racial representativeness of CCTs, including companion biobank participation, conducted in Cancer and Leukemia Group B (CALGB/Alliance Cooperative Group	Any, unspecified	Race/ethnicity SES	4
Hantel et al.	2024	Acute leukemia	Any/all	3,698 total patients	Evaluate racial and ethnic inequities in access to and enrollment in CCTs conducted at a comprehensive cancer center	Therapeutic, unspecified	Race/ethnicity SES	5
Hanvey et al.	2022	Gynecologic, gastrointestinal, thoracic	Any	692 total approached	Evaluate demographic and socioeconomic inequities in psychosocial CCT interest, eligibility, decline, enrollment, and retention	Psychosocial/ behavioral	Age Race/ethnicity SES	5
Hennessy et al.	2022	Gastro-esophageal	Metastatic	66 trials	Evaluate age-related representativeness and associated exclusion criteria in metastatic gastroesophageal CCTs from 1995 to 2020	Therapeutic, systemic, phase III	Age	5
Hori et al.	2007	Leukemia, lymphoma, esophageal, stomach, intestinal, liver, pancreatic, lung, breast, prostate, head and neck, uterus	Any	68 trials	Evaluate inequities in age between the Japanese cancer population and patients enrolled in new drug application (NDA) clinical trials	Drug, therapeutic	Age	5
Housri et al.	2015	Breast	*In situ* – II	264 total patients	Identify patient and tumor traits predicting HBRT enrollment among breast cancer patients	Radiotherapy	Race/ethnicity Sex Ability	5
Huang, Ezenwa, Wilkie, and Judge	2013	Any	Any	1,464 total patients 612 eligible patients	Assess sex and racial/ethnic differences in referral, eligibility, enrollment, and retention in two CCTs focused on pain and/or fatigue	Psychosocial/ behavioral	Race/ethnicity Sex	4
Hue et al.	2022	Pancreatic	Any	1,110 CCT enrollees 261,483 total	Evaluate demographic and clinical representativeness of pancreatic CCTs and associated survival	Any, unspecified	Race/ethnicity Age SES Sex Ability and comorbidity	5
Jan et al.	2022	Primary liver	Any	9749 CCT participants (63 CCTs)	To describe racial, ethnic, sex, and age representativeness of primary liver CCTs across the globe	Therapeutic, unspecified	Race/ethnicity Age Sex	5
Javid et al.	2012	Breast	I–IV	1,079 patients	Evaluate (1) age-related differences in CCT availability, eligibility, and enrollment and (2) patient- and physician-perceived barriers and facilitators in breast CCTs	Therapeutic, systemic	Age Ability and comorbidity	5
Javier-DesLoges et al.	2022	Breast, colorectal, lung, prostate	Any	242,720 CCT participants	Examine racial, ethnic, sex, and age representativeness of NCI CCTs and associated change across time	Any	Race/ethnicity Sex Age	5
Jayakrishnan et al.	2021	Any	Any	261 (total CCTs) 223 (CCTs reporting race)	Evaluate age and racial/ethnic representativeness and reporting patterns of FDA CCTs	Drug, therapeutic	Race/ethnicity Age	4
Kaanders et al.	2022	Head/neck	Any	87 RCTs	Evaluate the representativeness of head and neck cancer RCTs relative to the clinically treated population	Systemic, radiotherapy, surgical, hypothermic	Age Ability and comorbidity	5
Kanapuru et al.	2023	Multiple myeloma	Any	9325 CCT participants (16 CCTs)	Evaluate racial and ethnic disparities in eligibility and enrollment for multiple myeloma drug CCTs	Drug, therapeutic	Race/ethnicity	4
Kanarek et al.	2010	Brain, breast, gastrointestinal, hematopoietic, prostate, upper aerodigestive, viral/other	Any	5,068 accruals 17,637 total patients	Examine race/ethnicity and geographic location of residence on CCT enrollment at JH-SKCCC	Therapeutic, non-therapeutic	Race/ethnicity Geography	5
Keegan	2023	breast	Any	98 CCTs	Evaluate longitudinal change in racial reporting and representation in breast CCTs	Any, unspecified	Race/ethnicity (reporting)	5
Khadraoui et al.	2023	Endometrial, ovarian, cervical	Any	548 (CCT participants) 562,592 (total patients)	Evaluate racial and ethnic representativeness of gynecologic CCTs accounting for other demographic and socioeconomic covariates	Any, unspecified	Race/ethnicity Age SES Ability and comorbidity	4
Kilic et al.	2023	Lung	Any	311 (total CCTs) 9,869 participants for analysis (136 CCTs reporting race/ethnicity) 9,869 participants for analysis (136 CCTs reporting race/ethnicity)	Evaluate racial, ethnic, sex, and age representativeness of lung CCTs	Any, explicit supportive care inclusion	Race/ethnicity Sex Age	5
Ko et al.	2015	CNS, breast, GI, genitourinary, head and neck, lung, other	Any	99 trials 847 total screens	Identify characteristics of baseline eligibility, enrollment rates, reasons for ineligibility, and reasons for non-enrollment across CCTs	Therapeutic, non-therapeutic (explicit inclusion of supportive care)	Race/ethnicity SES	4
Kwak et al.	2023	Lung	Any	1924 CCT enrollees 1.6 million total patients	Evaluate racial, ethnic, and socioeconomic representativeness of lung CCTs	Any	Race/ethnicity SES	3
Ladbury et al.	2022	Breast, cervical, prostate, uterine	Any	77 trials (13,580 participants)	Evaluate age, racial, and ethnic representation in CCTs involving brachytherapy	Therapeutic, brachytherapy	Age Race/ethnicity	5
Langford et al.	2014	Any (primary: breast, colorectal, genitourinary)	Any	4509 patient logs	Evaluate racial and ethnic differences in CCT enrollment, refusal, eligibility, and desire to participate	Any, explicit supportive care (i.e., symptom management) inclusion	Race/ethnicity Age Sex Ability and comorbidity	5
Lythgoe, Savage, and Prasad	2021	Prostate	Any	18,455 CCT participants (17 CCTs, 9 reporting race)	Evaluate racial representativeness and associated reporting in FDA drug approvals for prostate CCTs	Drug, therapeutic	Race/ethnicity	5
Mishkin, Minasian, Kohn, Noone, and Temkin	2016	Gynecologic (cervical, ovarian, uterine)	Any	156 trials 18,913 accruals	Examine sociodemographic differences between NCI gynecologic CCT enrollees and incident gynecologic cancer population in the US	Therapeutic, unspecified	Age Race/ethnicity SES	4
Moloney and Shiely	2022	Breast	Any	40 CCTs	Assess demographic and socioeconomic inequities in breast CCT participation due to direct and indirect impact of eligibility criteria	Drug, therapeutic, phase III	Race/ethnicity Age SGM SES Geography Ability and Comorbidity	3
Murthy, Krumholz, and Gross	2004	Breast, lung, colorectal, prostate	Any	75,215 accruals	Compare CCT enrollees with population-based incidence data on age, sex, race, and ethnicity Determine whether size of sociodemographic inequities varied by age group or cancer type Determine whether racial/ethnic minority representation in CCTs has changed over time (1996–1998 compared to 2000–2002)	Therapeutic, nonsurgical	Age Race/ethnicity Sex	5
Newman et al.	2004	Breast, thoracic, gastrointestinal	All	7 CCTs	Evaluate sociodemographic accrual trends in ACOSOG CCTs	Surgical	Age Race/ethnicity	4
Noor et al.	2013	Any	Any	430 referrals 174 CCT accruals 10,784 population controls	Examine the effects of SES on the likelihood of referral to phase I CCTs and of enrollment	Any, unspecified, phase I		5
Osann et al.	2011	Cervical	I–III	380 recruitment letters 50 accruals	Use population-based data to identify disparities in accrual and retention of minority and/or low-income patients in a biobehavioral CCT	Psychosocial/ behavioral	Ethnicity SES Sex	3
Owens-Walton et al.	2022	Prostate, kidney, bladder/urothelial	Any	341 CCTs 49,202 CCT enrollees (of 169 CCTs reporting race)	Evaluate minority representativeness of urologic CCTs	Therapeutic, phase II and III	Race/ethnicity	4
Palmer et al.	2021	Prostate	I–II	855 total patients	Evaluate demographic and socioeconomic representativeness of various types of prostate CCTs based on self-report	Any, explicit behavioral inclusion	Race/ethnicity Age SES Ability and comorbidity	3
Pang et al.	2016	Lung (NSCLC, SCLC)	Any	131 trials 23,006 accruals 578,476 population controls	Identify inequities in CCT enrollment across age, race, ethnicity, and sex	Therapeutic, unspecified	Age Race/ethnicity Sex	5
Patel et al.	2020	Breast	0–II	2,472 invited patients	Investigate predictors of invitation to and participation in CCTs	Surgical, hormonal, systemic chemotherapy, radiation	Age Race/ethnicity Sex SES Ability	4
Patel et al.	2023	Gastrointestinal, head/neck	Any	1,446 total	Evaluate sociodemographic disparities in CCT eligibility and enrollment	Any	Race/ethnicity Sex Age SES Ability and comorbidity	5
Patki et al.	2023	Prostate	Any/all	138 full-text studies total 54 full-text studies reporting on EDI variables (19,039 participants)	Evaluate racial, ethnic, educational, and socioeconomic representativeness of treatment prostrate CCTs and associated reporting in manuscripts	Therapeutic, unspecified	Race/ethnicity SES	5
Perni, Moy, and Nipp	2021	Any	Any	2657 CCTs	Evaluate the sociodemographic and clinical representativeness of phase I CCTs, relative to that of phase II and III CCTs	Any, phase I–III	Race/ethnicity Sex Age SES	4
Pirl et al.	2018	Any	Any	18 total CCTs (3,960 patients) 10 patient CCTs reporting race/ethnicity (1,910 patients)	Evaluate racial and ethnic representativeness of and associated reporting practices for integrated palliative care CCTs	Supportive (palliative) care oncology	Race/ethnicity	5
Pittel et al.	2023	Lung, colorectal, breast, pancreatic, multiple myeloma	Advanced/metastatic	50,411 patients total (800 care sites)	Evaluate recent racial and ethnic representativeness of US CCTs in context of pre- and per-COVID-19 pandemic conditions	Drug, therapeutic	Race/ethnicity Age Sex Ability and comorbidity	4
Ramamoorthy et al.	2018	Breast, colorectal, lung, prostate	Any	2008–2013: 158 CCTs; 22,481 enrollees 2014–2017: 9 CCTs; 3,612 enrollees	Evaluate age, sex, racial, and ethnic representativeness of new oncologic FDA-approved products	Drug, therapeutic	Race/ethnicity Age Sex	5
Reihl et al.	2022	Glioma	Any	49,907 CCT participants (662 CCTs)	Evaluate racial, ethnic, and sex representativeness of CNS CCTs since NIH Revitalization Act	Therapeutic, phase I–IV	Race/ethnicity Sex Race and sex reporting	5
Riaz et al.	2023	Prostate	Any	104,205 (total CCT participants, global from 286 CCTs) 9,552 CCT participants (race-reporting CCTs in the USA)	Evaluate age, racial, and ethnic representativeness of prostate CCTs	Any, unspecified	Age Race/ethnicity Age, race, and ethnicity reporting	5
Saphner et al.	2021	Any	Any	39,968 total patients	Evaluate demographic and socioeconomic representativeness of CCTs	Any, unspecified	Race/ethnicity SES Age Sex	5
Sawaf et al.	2023	Rectal	Any	50 CCTs	Assess demographic and socioeconomic representativeness of US colorectal CCTs	Therapeutic, varied	Age Race/ethnicity Sex SES	5
Scalici et al.	2015	Cervical, endometrial, ovarian, sarcoma	Any	445 GOG studies 170 GOG studies reporting race 67,568 accruals 45,259 accruals reporting race	Determine minority participation proportions in GYN Oncology Group (GOG) CCTs	Any, phase I–III, observational, translational	Race/ethnicity	4
Sedrak et al.	2022	Any	Any	2,298 patients offered CCT	Evaluate age-related enrollment, ineligibility, and decline patterns in CCT relative to community cancer population	Any, explicit “non-therapeutic” inclusion	Age Ability and comorbidity	5
Shah et al.	2022	Melanoma	Any	20,912 CCT participants (35 CCTs)	Evaluate the sociodemographic representativeness of melanoma CCTs conducted in Europe, New Zealand, and Australian, with a focus on age	Therapeutic, phase III	Age	5
Shinder et al.	2023	Renal	I–IV	681 CCT participants 3,405 matched controls	Evaluate predictors of renal CCT participation	Any, unspecified	Age Race/ethnicity SES Sex Ability and comorbidity	5
Steventon et al.	2024	Gynecologic	Any	17,041 CCT participants (26 RCTs)	Evaluate racial and ethnic representativeness of gynecologic CCTs on US and global scale	Systemic therapies	Race/ethnicity (and reporting) Continental origin	5
Stewart, Bertoni, Staten, Levine, and Gross	2007	Breast, colon, lung, prostate	Any	13,991 accruals	Examine demographic characteristics of surgical CCT enrollment	Surgical	Age Race/ethnicity Sex	4
Talarico, Chen, and Pazdur	2004	Breast, lung, colorectal, ovarian, pancreatic, CNS, leukemia, lymphoma	Any	55 registration trials (28,766 patients)	Evaluate age representativeness of CCTs registering new cancer drugs approved by the FDA from 1995 to 2002.	Drug, therapeutic	Age	4
Tharakan, Zhong, and Galsky	2021	Any	Any	35 cancer drug approvals (w/ race reporting) 16,685 CCT enrollees (49 CCTs) 21 cancer drug approvals (w/ race and location) 10,318 CCT enrollees (21 CCTs)	Evaluate relationships between racial representativeness of US and global CCTs	Drug, therapeutic	Race Race reporting	4
Unger et al.	2020	Bladder, breast, colorectal, gastroesophageal, gynecologic, head and neck, leukemia, liver, lung, lymphoma, melanoma, myeloma, pancreas, prostate, renal	Any	85 pharmaceutical company trials (46,513 patients) 273 SWOG trials (47,512 patients)	Evaluate racial representativeness of pharmaceutical company-sponsored drug CCTs relative to those sponsored by the NCI National Clinical Trials Network (NCTN) and to the US oncologic population	Drug, therapeutic	Race	5
Unger, Gralow, Albain, Ramsey, and Hershman	2016	Breast, colorectal, lung	Any	1,581 patients 1,262 patients with income data	Examine effect of income and other sociodemographic covariates in predicting prospective enrollment in CCTs	Any, unspecified	SES	5
Unger et al.	2013	Breast, colorectal, lung, prostate	Any	5,499 evaluable respondents	Evaluate socioeconomic and other demographic predictors of CCT enrollment, attitudes, and reasons for decline	Any, unspecified	SES Age Race/ethnicity Ability and comorbidity	5
VanderWalde et al.	2022	Any	Any	66,708 CCT enrollees (237 CCTs)	Evaluate underrepresentation of older adults in CCTs in context of trial characteristics	Therapeutic, any	Age	5
Wagar et al.	2022	Ovarian, fallopian, peritoneal	Any/all	15 CCTs (3,414 enrollees)	Evaluate racial and ethnic representativeness of phase II and III poly (ADP-ribose) polymerase (PARP) inhibitor CCTs for ovarian cancer	Therapeutic, PARP inhibitor	Race/ethnicity	4
Yekedūz et al.	2021	Solid tumors	Any/all	105,397 CCT enrollees (142 CCTs)	Evaluate sociodemographic inequities in CCT participation for solid organ tumor drug trials	Drug, therapeutic	Ability & comorbidity	5
Yonemori et al.	2010	CNS, oral/pharyngeal, lung, gastric, liver, gallbladder, colon, kidney, bladder, pancreas, skin, breast, uterine, ovarian, prostate, lymphoma, myeloma, leukemia	Any	234 trials	Evaluate older adult CCT participation for NDAs or extension of indications for oncology drugs or supportive care	Drug, therapeutic, explicit supportive care inclusion, phase I	Age Ability	5
Zafar et al.	2011	Any	Any	216 patients	Describe sociodemographic, disease, treatment characteristics of older patients presenting to Phase I Clinical Trial service	Drug, therapeutic	Age Ability	3
Zhao et al.	2024	Breast, prostate, colorectal, lung	Any	7747 total CCTs	Evaluate the sociodemographic representativeness of common CCTs, with a focus on older adults	Therapeutic, phase III	Age	5
Zullig et al.	2016	Lung, colorectal, breast, prostate	Any	13,795 accruals 588,317 incident cases	Evaluate sociodemographic characteristics of CCT enrollment in North Carolina	Therapeutic, unspecified	Race/ethnicity	4
Zuniga et al.	2020	Prostate	Localized, Advanced	26 trials 2316 accruals 608,006 incident cases	Describe reporting of race and race-specific analyses of Black prostate cancer patients in lifestyle intervention CCTs Evaluate distribution of Black patients in lifestyle CCTs compared to Black patients with prostate cancer in the USA	Psychosocial/ behavioral	Race Sex	4

*Note:* Abbreviations included in this table are utilized as follows, listed alphabetically: ACOSOG = Alliance for Clinical Trials in Oncology; CALGB = Cancer and Leukemia Group B; CAR-T = chimeric antigen receptor T-cell; CCC = comprehensive cancer center; CCT = cancer clinical trial; EDI = Equity, Diversity, and Inclusion; FDA = Food and Drug Administration; HBRT = hypofractionated breast radiotherapy trials; HPV = human papillomavirus; JH-SKCCC = Johns Hopkins Sidney Kimmel Comprehensive Cancer Center; NCDB = National Cancer Database; NCI = National Cancer Institute; NIH = National Institutes of Health; NR = not reported; NSCLC = non-small cell lung carcinoma; OPSCC = oropharyngeal squamous cell carcinoma; SES = socioeconomic status.

**Table 2.** Methodology of studies

Authors	Year	Study design	Recruitment	Databases	Measures	Analysis
Abbas et al.	2022	Case-control study	Archival database	ClinicalTrials.gov, NCI Cancer Therapy Evaluation Program (CTEP, 2000–2019) National Cancer Database (NCDB, 2004–2017)	Patient: Dichotomized age (@65) Race/Ethnicity (NHW, NHB, AAPI, H) Insurance coverage Cancer site Residential ZIP code (median household income, HS educational attainment) Institutional: CCT slot Facility location Enrollment fraction (EF)	Preliminary chi-square and t-tests Multivariate logistic regression
Abi Jaoude et al.	2020	Meta-analysis	Archival database	ClinicalTrials.gov	Presence of exclusionary criteria Performance status: ECOG score	Chi-square tests Binary logistic regression
Acoba, Sumida, and Berenberg	2022	Case-control study	Archival database	UHCC OnCore Hawaii Tumor Registry	Race (White, Chinese, Filipino, Japanese, Native Hawaiian) EF	Non-parametric descriptive inferential testing
Ajewole et al.	2021	Cohort study	Archival database	FDA Hematology/Oncology Approvals (2009–2019)	Race reporting Race (and ethnicity): White, Asian, Black, Hispanic	Descriptive statistics
Al Hadidi et al.	2022	Case-control study	Archival database	Drugs@FDA (CAR-T therapies 2017–2021)	Enrollment proportion Prevalence statistics (from DeSantis et al. (2019))	Participant-to-prevalence ratios
Aldrighetti et al.	2021	Meta-analysis	Archival database	ClinicalTrials.gov (through April 2021) Surveillance, Epidemiology, and End Results (SEER) US Census	Race/ethnicity (NHW, *B*, AAPI, AI/AN, H) O:E ratios for enrollment relative to incident population	Meta-Analysis of O:E enrollment ratios
Awad et al.	2020	Meta-analysis	Literature search, archival database	PubMed (1985–2018), US CDC (1999–2015) CDC age-adjusted incidence	Age Race Tumor type Publication year Age-adjusted incidence by type (US CDC) Expected enrollment ratio (White [W]:Black [B]) Expected:Observed ratios	T-tests Chi-square tests ANOVAs
Baldini et al.	2022	Case-control study	Archival database, internal clinical infrastructure	EGALICAN-2 survey (11 early-phase units) GLOBOCAN	Population-based incidence rates (2020)	Preliminary chi-square and Fisher’s exact tests One sample z-test Logistic regression
Baquet, Ellison, and Mishra	2009	Case-control study	Archival database	Cancer Therapy Evaluation Program (NCI CTGC, 1999–2002) Maryland Cancer Registry (1999–2002)	Patient level : Cancer site 10-year age group, Race (W, *B*, Other) Sex Residential ZIP code Insurance status (private, Medicaid or Medicare, self-pay, military) County level: Material deprivation (%poverty, households w/o car, 16+ unemployed, owner-unoccupied housing) Social class (% 25+ HS graduates, grad/professional degrees, white-collar occupations, median household income, population composition) Urban/rural (Beale classification system)	Preliminary chi-square tests Logistic regression
Behrendt, Hurria, Tumyan, Niland, and Mortimer	2014	Cohort study	Internal treatment center, archival database	City of Hope Comprehensive Care Center (2004–2009) US Census Bureau American Community Survey (2007–2011)	Primary: birthplace/race/ethnicity (African, Asian, Latin American, Eastern European, Middle Eastern, Other Caucasian) Accrual status Covariates Patient level Primary language Tumor (stage, HR status, HER2/neu status, year of first visit, time since Dx) Oncologist level PI status Duration of practice Linguistic fluency ZIP code level Median household income (12 months) % w/o HS education among 25+ women	Preliminary bivariate correlation Logistic regression
Bero et al.	2021	Case-control study	Archival database	ClinicalTrials.gov (1996–2019) US Census (2018)	EF	Chi-square analysis
Borad et al.	2020	Cohort study	Archival database	ClinicalTrials.gov (8/2000–2/2020)	Mean and median age Trial treatment type Trial country	Descriptive statistics
Borno et al.	2019	Case-control study	Internal treatment center, archival database	CTMS, UCSF, Helen Diller Family CCC California Cancer Registry, UCSF catchment area (2010–2014)	Dichotomized age (@65) Race/ethnicity (W, *B*, AAPI, Latino, Other)	Chi-square tests
Brierley et al.	2020	Case-control study	Archival database	US MDS CRC (1991–2017, data from 5/6 institutions) SEER-Medicare International Working Group for Prognosis in MDS	Age Sex Race/ethnicity Distance to treatment center Blood counts and creatinine MDS subtype ECOG PS Therapy-related disease Zip code (income proxy: total income/# inhabitants)	Preliminary Kruskal–Wallis tests, Chi-square or Fisher’s exact test Logistic regression
Bruno, Li, and Hess	2024	Cohort study	Archival database	Merative MarketScan Medicaid claims database (2017–2019)	Race (W, *B*, Other) Age Sex Staging CCT participation likelihood	Preliminary chi-square and t-tests Logistic regression
Bruno et al.	2022	Cohort study	Archival database	Flatiron Health Electronic Health Record (2011–2017)	Age Race (W, *B*, Asian, Other, Unknown) Ethnicity (NH, H) Stage Insurance Functional status (ECOG) Cancer covariates Institutional covariates CCT participation (use of the clinical trial drug during the period of observation)	Preliminary chi-square analyses Stepwise linear regression
Canoui-Poitrine et al.	2019	Cohort study	Archival database	*Sujets AGes dans les Essais Cliniques* (SAGE; Older Subjects in Clinical Trials, 2012–2016)	Age: 65–69, 70–74, 75–79, 80+ Sex Disease site and stage Performance status Comorbidity MMSE, NCD history, ADL score, mini-GDS, polymedication, incontinence, mini-Nutritional Assessment Test Trial sponsor, phase, treatment CCT eligibility, invitation, reasons for ineligibility, non-invitation, non-inclusion	Chi-square, Fischer, and Kruskal–Wallis tests Multivariate logistic regression Multivariate logistic regression
Casey et al.	2023	Case-control study	Archival database	FDA “Oncology/Hematologic Malignancies approval notifications” (2011–2021) FDA “Novel Drug Approvals” (2011–2021) SEER Explorer (2014–2018) County Health Rankings and Roadmaps Small Area Health Insurance Estimates (2020)	Race (AI/AN, AAPI, *B*, W, Oth) Ethnicity (NH, H) Sex Age ZIP Code (CCT representation fraction relative to population burden estimates	Chi-square and Fisher’s exact tests
Choradia et al.	2024	Case-control study	Archival database	Biomedical Translational Research System (BTRIS, 2005–2020) SEER (2018) Cancer in North America (CiNA) database (2018) North American Association of Central Cancer Registries	Dichotomized age (@65) Race (W, *B*, AAPI, AI/AN, multiracial, unknown) Ethnicity (NH, H) Sex State Country Enrollment fraction	Preliminary chi-square tests Logistic regression
Costa, Hari, and Kumar	2016	Meta-analysis	Literature search, archival database	PubMed (2007–2014) SEER-18 ISS (1981–2002) Mayo (2001–2010)	Study level : Study phase Tx status Study size Sponsor type Patient level: Age Sex Stage Race/ethnicity (dichotomized NHW vs. racial and/or ethnic minority)	Preliminary chi-square and Fisher’s exact tests, Mann–Whitney tests Byar approximations for ratios
Craig, Gilbery, Herndon, Vogel, and Quinn	2010	Case-control study	Archival database	SEER-Medicare (Sep 2000–Dec 2002)	SES: Median income using IQR of zip code to categorize neighborhoods into low, middle, high; ZIP codes US Dept. Agricultural rural–urban continuum Census region: Northeast, South, Midwest, West Tumor characteristics: grade, PSA status, stage Race/ethnicity: W, *B*, Hispanic, Other Education: < HS, HS, some college, college graduate	Preliminary Wilcoxon–Mann–Whitney U, chi-square tests Logistic regression
Diehl et al.	2011	Cross-sectional study	Archival database	ACOSOG trials (1999–2009)	Race/ethnicity via patient report at trial registration Predictors: randomization, staging (early vs. advanced), design (drug vs. surgical) Success level: successful, modestly successful, unsuccessful measured by the proportion of AA and HA participants based on general and oncologic population characteristic ASOSOG recommendations for accrual targets: early-stage breast: AA 11+%. HA 5+% regionally advanced breast: AA 14+%, HA 5+% Non-metastatic lung: AA 10+%, HA 2+% Metastatic lung: AA 12+%, HA 2+%	Proportions relative to the general population and oncologic population
Dressler et al.	2015	Cohort study	Archival database	Cancer and Leukemia Group B (Alliance for Clinical Trials in Oncology, after 2003) Alliance Statistics and Data Center Clinical Trials Support Unit	Patient characteristics: Age Sex Race (dichotomized) Cancer type Institutional characteristics : Site registration Accrual patterns Accrual patterns specific to pharmacogenomic component Exploratory institutional diversity via minority participation fraction Probability of consent to pharmacogenomic studies	Preliminary chi-square and Wilcoxon rank-sum tests Logistic regression
Du, Gadgeel, and Simon	2006	Cohort study	Internal treatment center, archival database	Karmanos Cancer Institute (Jan 1, 1994–Dec 31, 1998) SEER (MDCSS)	Sex Age at Dx Race dichotomized Insurance coverage (commercial, Medicaid, Medicare only, Medicare Plus) SES rank Stage Histology SWOG PS Comorbidities	Preliminary chi-square and t-tests Logistic regression
Dudipala et al.	2023	Cohort study	Archival database	Electronic Medical Records (EMRs) (1/2015–12/2020)	Age Race Ethnicity Sex City Primary language Median household income Insurance Education Stratified proportion CCT discussed Stratified proportion CCT enrolled	Preliminary chi-square and Fisher’s exact tests Multivariate logistic regression
Duma et al.	2018	Meta-analysis	Archival database	ClinicalTrials.gov (2003–2016) SEER (2013)	Race/ethnicity Sex EF Race/ethnicity reporting Time period (1996–2002, 2003–2016) Race/ethnicity reporting Time period (1996–2002, 2003–2016)	Chi-square tests
Earl et al.	2023	Case-control study	Archival database	Huntsman Cancer Institute (HCI) Research Informatics Shared Resource (May 2012–May 2022) HCI Clinical Trial Office Utah Cancer Registry (Jan 2010–Dec 2019)	Rurality (county: frontier, rural, urban) Household per capita income (2019) County % HS education+ County glioma incidence estimates Enrollment fraction	One-way ANOVA (Tukey post hoc) Chi-square analysis
Elshami et al.	2022	Cohort study	Archival database	NCDB (2004–2017)	Age Sex Race Ethnicity Education Median income Insurance (primary) Facility type, distance Staging Comorbidity score Histology Rate of CCT enrollment	Preliminary chi-square tests Multivariate logistic regression
Eskander et al.	2022	Cohort study	Archival database	NCDB	Age Race Ethnicity ZIP-code median income and %HS edu Insurance coverage Facility distance, type, and location Charlson-Deyo comorbidity score	Preliminary chi-square and Wilcoxon rank sum tests Multivariate logistic regression
Fakhry et al.	2023	Meta-analysis	Archival database	PubMed, Embase, World of Science, Cochrane (through 7/27/2021); US Census Data (2020)	Race/ethnicity report Race/ethnicity representation (W, *B*, AI/AN, Asian, NH/PI, Multi, H) Population-based incident estimates	Descriptive proportions
Fayanju et al.	2019	Case-control study	Archival database	NCI Cancer Therapy Evaluation Program (CTEP) National Cancer Database (1998–2012) ClinicalTrials.gov (2000–2012)	Age at Dx (<40, 40–64, 65+) Year of enrollment Race/ethnicity (NHW, NHW, API, Hispanic, Native American, Other) ZIP-code level Median household income % HS graduates Enrollment decision	Preliminary chi-square and t-tests Logistic regression
Freudenburg et al.	2022	Meta-analysis	Archival database	MEDLINE (1/1/1970–2/29/2020) Clinicaltrials.gov (1997–2020)	Race reporting (C, AA, Other, Asian, *H*, NA)	Descriptive proportions Qualitative synthesis
Gopishetty, Kota, and Guddati	2020	Case-control study	Archival database	NIH trials (Jan 1, 1999–Jan 1, 2019) US Cancer Statistics	Age- and race-adjusted incidence by type CCT enrollment	Chi-square tests
Gordis et al.	2022	Meta-analysis	Archival database	PubMed, Scopus, CINAHL, Cochrane Library (through 2/2/2022) NCDB	Age Sex Race Cancer history Tumor site Behavioral health history (smoking, alcohol)	Meta-analysis with Freeman-Tukey weighted-summary proportion
Grant et al.	2020	Case-control study	Archival database	ClinicalTrials.gov SEER (dates unspecified, 5-year increments)	Difference in incidence by race/ethnicity between CCT and SEER incident cases Ratio of incidence by race/ethnicity via median ratio of CCT and SEER incident cases	Preliminary Mann–Whitney U and Kruskal–Wallis ANOVAs Wilcoxon signed-rank test for comparing D-IRE to 0 and R-IRE to 1
Green et al.	2022	Cohort study	Archival database	Medicare FFS claims data Clinicaltrials.gov (1/1/2015–6/30/2020)	Dichotomized age (@65) ZIP code (median income) CCT enrollment	Descriptive proportions
Grette et al.	2021	Case-control study	Archival database	Clinicaltrials.gov CDC (age-adjusted rates)	Race reporting Participant race (W, *B*, Asian, Other) Tumor site Age-adjusted incidence rates	Chi-square analyses
Gross, Filardo, Mayne, and Krumholz	2005	Case-control study	Archival database	NCI CTEP (1996–2001) SEER-Medicare	SES: % below poverty level (zip), % unemployed (county), insurance (private, Medicare, Medicaid, Medicare + private, Medicare + Medicaid, VA, self-pay, uninsured, other) Age: 65–59, 70–74, 75–79, 80–84, 85+ Race/ethnicity: W, NHB, Hispanic, API Distance between home and site	Preliminary chi-square and t-tests Logistic regression
Guerrero et al.	2018	Meta-analysis	Archival database	PubMed	Presence of race/ethnicity reporting Race/ethnicity	Descriptive statistics
Hantel et al.	2024	Cohort study	Archival database	Dana-Farber/Harvard Cancer Center (DF-HCC) cancer and clinical trials registries Massachusetts Cancer Registry (MCR) 1/1/2010–12/31/2019	Race/ethnicity (NHW, NHB, NHA, HW, Other) Insurance Marital status Driving distance State Yost Index (sYI) Age Sex Subtype Access (treatment at a DF-HCC hospital) CCT enrollment	Preliminary chi-square, Fisher’s exact, and Kruskal–Wallis tests Multivariate logistic regression
Hantel et al.	2022	Cohort study	Archival database	CALGB/Alliance Statistics and Data Management Center (through 8/26/2021; enrollment 1998–2013) SEER and 2010 US Census data	Race/ethnicity SES Age Sex ZIP-code Consent forms Enrollment fraction Incidence estimates	Preliminary chi-square, Fisher’s exact, and Wilcoxon rank-sum tests Multivariate logistic regression
Hanvey et al.	2022	Cohort study	Archival database	CBTi.p. intervention (2009–2017) Yoga intervention (2017–present)	Dichotomized age (@60) Dichotomized race, ethnicity, and racial/ethnic minority status SES composite (income, education, employment 0–7) Dichotomized rurality (large metro vs. other) Psychosocial symptom scores: BDI-II, STAI, MPQ, PSQI Eligibility Decline Reasons for decline Eligible enrollment Voluntary attrition/death	Preliminary chi-square tests Logistic regression Survival analysis with GDTMs
Hennessy et al.	2022	Meta-analysis	Archival database	Embase, PubMed, Cochrane Library (1/1/1995–11/18/2020)	Age Age restriction Study location Time (10-year period)	Binary logistic regression
Hori et al.	2007	Case-control study	Archival database	Review reports submitted as NDA trials from Pharmaceuticals and Medical Devices Agency (Sep 1999–Apr 2005) Cancer Statistics in Japan (2003)	Median age (or mean of median group ages) across entire enrolled CCT samples calculated Age-specific incidence rates by cancer type	Comparisons of median ages between patient population and CCT accruals by type (unspecified)
Housri et al.	2015	Cohort study	Internal treatment center, archival database	Rutgers Cancer Institute of New Jersey (Jun 2009–Dec 2012)	Demographics, stage, grade, receptor status, family history of breast cancer in 1st degree relative, radiation dose, concurrent Tx, site of initial consultation	Preliminary chi-square or Fisher’s Exact Logistic regression
Huang, Ezenwa, Wilkie, and Judge	2013	Cohort study	Internal treatment center, ongoing database	“ResearchTracking” (University of Washington Cancer Center, Seattle Cancer Care Alliance)	Age Sex Eligibility status Reasons for ineligibility Enrollment status Completion status Withdrawal reasons	ANOVAs, Fisher’s exact tests
Hue et al.	2022	Case-control study	Archival database	NCDB (2004–2016)	Race (NHW, NHB, Other) ZIP-code median income, %HS edu Age Sez Insurance primary Charlson-Deyo score Stage	Preliminary chi-square and Wilcoxon rank-sum tests Multivariable logistic regression Kaplan-Meier and Cox regression survival analyses
Jan et al.	2022	Meta-analysis	Archival database	Clinicaltrials.gov (through 7/19/2019) US Cancer Statistics database GLOBOCAN	Race (W, *B*, AAPI, AI/AN, multi, unknown) Ethnicity (NH, H) Dichotomized age (@65) Sex EF	Chi-square and Fisher’s exact tests
Javid et al.	2012	Cross-sectional study	Multiple	NR (survey administration)	Demographics: marital status, education, travel, transportation, income Patient Participation/Refusal Questionnaires (reasons) Reasons for ineligibility Trial availability, eligibility, and enrollment	Chi-square tests Logistic regression
Javier-DesLoges et al.	2022	Cohort study	Archival database	NCI Clinical Data Update System (2000–2019) Cancer Incidence Data (CDC US Cancer Statistics, 2000–2017)	Race/ethnicity (W, *B*, H, AAPI) Sex Age Diagnostic site Incident population values	Multivariate logistic regression
Jayakrishnan et al.	2021	Case-control study	Archival database	FDA drug approvals (7/2007–6/2019) cancer.org, seer.cancer.org (8/1/2020)	Age Race (reporting)	Chi-square tests, t-tests, MANOVAs
Kaanders et al.	2022	Meta-analysis	Archival database	MEDLINE, Epub Ahead of Print, Embase, Cochrane Central Register of Controlled Trials, ClinicalTrials.gov (2009–2019)	Age Performance status Recruitment rate	Chi-square and Mann–Whitney U
Kanapuru et al.	2023	Case-control study	Archival database	FDA drug approvals (2006–2019)	Race (W, *B*, Asian, NH/PI, AI/AN, Other, Unknown) Ethnicity (NH, *H*, Unknown) Age (<65, 65–75, 75+ Sex Country Eligibility Reasons for eligibility Enrollment	Pooled descriptive statistics
Kanarek et al.	2010	Case-control study	Internal treatment center, archival database	Johns Hopkins Cancer Registry JH-SKCCC Clinical Research Office (2005–2007)	Accrual to cancer case ratio (ACR) to determine ACR “relative risk” for each demographic subgroup to reference group Place of residence via zip codes: Baltimore City, non-Baltimore City catchment area, non-catchment area Race: White, Black, other (including Hispanic individuals) Covariates: age (<20, 20–64, >64), sex, county poverty level (% of individuals at or below poverty in 2003), cancer site (high: hematologic, medium: prostate and gastrointestinal, low: other)	Preliminary ANOVAs Poisson regression VIF statistic (multicollinearity SES, race)
Keegan et al.	2023	Cohort study	Archival database	Clinicaltrials.gov	Year Reporting quarter Race reporting Quarterly trend in race reporting proportion	Frequencies and proportion with SE and CIs Linear regression
Khadraoui et al.	2023	Cohort study	Archival database	SEER, NCDB (2004–2019)	Race/ethnicity (W, *B*, H, Asian, NH/PI, AI/AN) Age Insurance Charlson-Deyo comorbidity Area SES (income, % without HS educational, metropolitan status) Facility variables: location, type Clinical variables: stage, treatment history, grade CCT enrollment Participation-to-prevalence ratios	Multivariate logistic regression
Kilic et al.	2023	Case-control study	Archival database	US National Library of Medicine / ClinicalTrials.gov (2004–2021) SEER	Race/ethnicity (and reporting; NHW, NHB, NHAPI, NHAIAN, NHUR, Hispanic) Dichotomized age (@65) Sex Enrollment	t-tests, Kruskal–Wallis tests Multivariate logistic regression
Ko et al.	2015	Cohort study	Internal treatment center, archival database	Boston Medical Center Clinical Trials Office: BMC Cancer Center (Jan 1, 2010–Dec 31, 2010)	Sociodemographic (EMR): age, race/ethnicity, sex, employment, primary spoken language, country of birth, primary insurance, highest education level, marital status Eligibility: dichotomized Ineligibility reason further dichotomized: no open trial vs. not eligible for open trial Enrollment: dichotomized Non-enrollment reason further dichotomized (patient vs. provider decline)	Chi-square and t-tests
Kwak et al.	2023	Case-control study	Archival database	NCDB (2004–2018)	Race/ethnicity (NHW, NHB, H) Facility type Insurance coverage	Preliminary chi-square and Wilcoxon rank sum Multivariate logistic regression Kaplan-Meier survival and Cox regression
Ladbury et al.	2022	Case-control study	Archival database	ClinicalTrials.gov (through 1/4/2020) SEER (2000–2016)	Age Race Ethnicity Cancer type Age reporting Race/ethnicity reporting Enrollment incidence disparity Enrollment incidence ratio (EIR)	T- and chi-square tests
Langford et al.	2014	Cohort study	Archival database	NCI Community Cancer Centers Program (NCCCP) Clinical Trial Screening and Accrual Log (3/2009–5/2012)	Demographic: race/ethnicity, age, sex, country region Consent length, readability CCT refusal, lack of desire to participate, enrollment, physical/medical conditions Consent length, readability CCT refusal, lack of desire to participate, enrollment, physical/medical conditions	Preliminary chi-square tests Binary logistic regression
Lythgoe, Savage, and Prasad	2021	Case-control study	Archival database	FDA licensing (1/2006–7/2020)	Race (W, *B*, Asian, AI/AN, Other/multiracial, unknown/missing) Race reporting	Descriptive proportions
Mishkin, Minasian, Kohn, Noone, and Temkin	2016	Case-control study	Archival database	CTEP Clinical Data Update Service (2003–2012) SEER (2003–2012) US Census (2010)	Demographic variables: race (American Indian, API, Black, White, unknown); ethnicity (Hispanic, non-Hispanic, unknown), age (time of trial registration), insurance (private, Medicaid, uninsured, unknown, 2007–2012; 65+ excluded due to Medicare) Population-based incidence: SEER incidence rates * 2010 Census population within each category	Did not use inferential statistics due to the use of complete accrual population Relative differences within subgroups assessed (5%+ differences considered clinically important)
Moloney and Shiely	2022	Meta-analysis	Archival database	MEDLINE (2010–2020)	Eligibility criteria imposed Clinical/scientific rationale for criteria imposed	Descriptive proportions Qualitative synthesis
Murthy, Krumholz, and Gross	2004	Case-control study	Archival database	CDUS (1996–2002) NCI PDQ Database of Clinical Trials (50 largest trials) SEER (1995–1999)	EF: # CCT enrollees/estimated # US cases (adjusted for age and racial/ethnic group) Race/ethnicity: Enrollees Cancer Therapy Evaluation Program categories (1996–2001) – White, Black, API, AI/AN, Hispanic; 2002 – Hispanic ethnicity as separate category for Population Data NHW, NHB, NH-API, NH-AI/AN, Hispanic Cancer incidence: rates determined for each 5-year age range, race, sex è # SEER cases/population SEER county è rates applied to US population	Chi-square tests Crude odds ratios Polytomous logistic regression Huber-White robust variance
Newman et al.	2004	Case-control study	Archival database	ACOSOG, SWOG, NCI (Oct, Nov 2003) SEER	Proportion by race, by age dichotomized at 65	Descriptive statistics (otherwise NR)
Noor et al.	2013	Case-control study	Internal treatment center, archival database	Thames Cancer Registry Guy’s Hospital phase I clinic	Patient data from referrals, notes, Rx records: age at referral, primary tumor, sex, ethnicity, postal code, dichotomized enrollment Population incident cases: TCR SES: Index of Multiple Deprivation: calculated from income, employment, health, education, crime, access, living environment scores assigned to geographic areas; patients assigned scores based on postal code	Preliminary crude odds ratios Logistic regression
Osann et al.	2011	Cross-sectional study	Community outreach	CSPOC, LACCSP cancer registries	Race/ethnicity: cancer registry; all non-Hispanic individuals grouped as 1 Enrollment/refusal rates	Chi-square tests Logistic regression MANOVAs
Owens-Walton et al.	2022	Cohort study	Archival database	ClinicalTrials.gov (2000–2017) SEER (2000–2017)	Race/ethnicity (W, *B*, AAPI, AI/AN, *H*, multi, unknown/NR) Representation quotient	Descriptive representation quotients
Palmer et al.	2021	Cross-sectional study	Archival database, mail, phone	California Cancer Registry	Race (African American, Asian American, Latino, White) Age (50–54, 55–64, 65+) Marital status Education Region Language Insurance Health literacy Health status Comorbidities endorsed Treatment history CCT participation (any, behavioral, bio/clinical, none)	Multivariate logistic regression
Pang et al.	2016	Case-control study	Archival database	NCI-sponsored cooperative groups trials (1990–2012) SEER (1990–2012)	Elderly = 70+ Enrollment disparity difference: absolute difference between est. group proportion in US lung cancer population and that of trial participants Enrollment disparity ratio: group proportion in US lung cancer population divided by that of trial participants Annual percentage of change (APC) in subgroup enrollment	APC Joinpoint regression
Patel et al.	2023	Cohort study	Archival database	University of Michigan Health Rogel Cancer Center clinical trials database EMR	Age Sex Race Marital/family status Employment Insurance Charlson Comorbidity Index Clinical factors: type, stage, histology CCT Eligibility Offered CCT CCT enrollment	Preliminary chi-square tests Multivariate logistic regression
Patel et al.	2020	Cross-sectional study	Archival database	iCanCare Study ClinicalTrials.gov SEER (Georgia, Louisiana, 2013–2014)	Age: −50, 51–65, >65 Comorbidities: 0 vs. 1+ Surgeries, chemo, radiation Stage (0–II) White, Black, Latina, Asian, Other/unknown Acculturation: high vs. low Marital status Education: −HS, some college, technical vs. college+ Income: <$40,000 vs. $40,000+ Insurance: none, Medicaid, other public, Medicare, private Geographic site Distance from treatment center: −30, 31+ Employment and flexibility (dichotomized) Decision-making style: 5-point Likert scale (intuitive to rational) Outcomes dichotomized	Preliminary chi-square tests Logistic regression
Patki et al.	2023	Meta-analysis	Archival database	CENTRAL, MEDLINE, Embase (through 2010–4/24/2020)	Race, ethnicity, SES, and educational attainment reporting Descriptive proportions of CCT participant race, ethnicity, SES Additional outcomes where reported: Age Stage SES group Education Eligibility criteria Study outcomes	Descriptive statistics and qualitative synthesis
Perni, Moy, and Nipp	2021	Cohort study	Archival database	Massachusetts General Hospital Cancer Center EHRs (10/1/2011–11/30/2014)	Race/ethnicity Sex Age Insurance status Marital status Income (median ZIP code) CCT phase I, II, and III enrollment	Preliminary chi-square and Wilcoxon rank-sum tests Multivariate logistic regression
Pirl et al.	2018	Meta-analysis	Archival database	2012, 2017 ASCO statements on palliative care in oncology PubMed	Race/ethnicity Race/ethnicity reporting Other demographic data reporting: age, sex, marital status, education, income, religion Trial setting Language eligibility requirements	Descriptive statistics
Pittell et al.	2023	Cohort study	Archival database	Flatiron Health Inc. (1/2017–12/31/2022)	Age Race/ethnicity (W, *B*, L) Cancer type Pre/post-COVID ECOG Region Practice type Sex CCT participation	Stratified hazard models
Ramamoorthy et al.	2018	Case-control study	Archival database	CDER, FDA (Drugs@FDA)	Race/ethnicity Age Sex Time period (2008–2013; 2014–2017)	Descriptive statistics (proportions)
Reihl et al.	2022	Meta-analysis	Archival database	PubMed (1/1/2000–12/31/2019) ClinicalTrials.gov Central Brain Tumor Registry of the US (CBTRUS (2000–2017) SEER-18 (2000–2017)	Sex Race/ethnicity (W, Asian, *B*, H) CCT enrollment Survival Stratified, population-based incidence and mortality rates	Chi-square and Fisher’s exact tests
Riaz et al.	2023	Meta-analysis	Archival database	MEDLINE (through 2/2021) Global Burden of Disease SEER-21 (2000–2018)	Dichotomized age (@65) Race/ethnicity (AA/B, AAPI, W) Outcomes using population-based incident estimates: Enrollment incidence ratios Demographic trial proportions	Meta-regression with random effects
Saphner et al.	2021	Case-control study	Archival database	Aurora Health Care Cancer Registry (8/1/2013–7/31/2019) American Community Survey (2014–2018)	Age Sex Race (W, *B*, Asian, NA/AN, HI/PI) Ethnicity Area SES -Median household income, standardized to range from 0 to 1 -Percentage of people below the federally defined poverty line -Median value of owner-occupied values, standardized to range from 0 to 1 -Percentage of people aged 16 years or older in the labor force who are unemployed (and actively seeking work) -Percentage of people aged 25 years or older with at least 4 years of college -Percentage of people aged 25 years or older with less than a 12th grade -Percentage of households containing one or more person per room CCT participation	Preliminary chi-square and Mann–Whitney tests Multivariate logistic regression
Sawaf et al.	2023	Meta-analysis	Archival database	PubMed (through 12/2019) NCDB (2010–2019)	Age Sex Race/ethnicity Rurality Facility type, location Demographic and socioeconomic reporting	Qualitative synthesis Chi-square and one-sample t-tests where quantification possible
Scalici et al.	2015	Case-control study	Archival database	GOG website (1985–2013 publications) CDC	Type of study CDC age-adjusted incidence for comparison between expected and observed cases by race (ratio W:B) Race/ethnicity: B, W Tumor site: ovary, endometrium, cervix, sarcoma Year published: 1993 and lower, 1994–2002, 2003–2008, and 2009–2013)	Chi-square and t-tests ANOVAs
Sedrak et al.	2022	Cohort study	Archival database	NCI Community Oncology Research Program (NCORP, 1/1/2016–12/31/2019)	Primary: Age Reasons for ineligibility Reasons for decline Enrollment Sociodemographic covariates: sex, marital status, ethnicity, SES indicators, comorbidity types	Chi-square analyses
Shah et al.	2022	Meta-analysis	Archival database	ClinicalTrials.gov	Weighted mean/median age	Weight mean/median calculation
Shinder et al.	2023	Case-control study	Archival database	NCDB (2004–2014)	Age Race/ethnicity Sex Insurance Stage Charlson-Deyo comorbidity Area median income Area % HS education Facility location, type CCT participation	Multivariate logistic regression
Steventon et al.	2024	Meta-analysis	Archival database	Licensed systemic anti-cancer therapies (1/11/2012–1/11/2022)	Race/ethnicity Continent	Descriptive statistics
Stewart, Bertoni, Staten, Levine, and Gross	2007	Case-control study	Archival database	NCI CDUS, NCI CTEP (2000–2002) SEER (2000–2002) US Census (2000)	Race/ethnicity: NHW, NHB, API, AI/AN, Hispanic from Census (2000) Age: 5-year intervals 20–74, 75+ from Census (2000) Cancer incidence rates: SEER EF: # enrollees/estimated # US cancer type cases	Logistic regression
Talarico, Chen, and Pazdur	2004	Case-control study	Archival database	FDA (1995–2002) SEER-11 (1995–1999)	Age: %65+, %70+, %75+	Chi-square tests
Tharakan, Zhong, and Galsky	2021	Case-control study	Archival database	FDA cancer drug approvals (2015–2018) American Cancer Society (2012–2016)	Racial enrollment distribution per CCT Geographic location per CCT Disparity score per CCT (#Black enrollees/US incidence per cancer)	Pearson correlation
Unger et al.	2020	Case-control study	Archival database	FDA drug approvals (2008–2018) NCTN data (SWOG Cancer Research Network) SEER	% Black race Trial sponsorship: pharmaceutical company, SWOG Cancer type	Tests of proportions
Unger, Gralow, Albain, Ramsey, and Hershman	2016	Cohort study	Internal treatment centers (8), archival database	NR	Age Race/ethnicity Sex Income (@ $50k) Education Distance from clinic Disease status	Logistic regression
Unger et al.	2013	Cross-sectional study	Community outreach	NexCura treatment decision tool	SES (income, education) Age Race Comorbidity score Discussion of CCT with provider CCT beliefs and attitudes CCT enrollment Discussion of CCT with provider CCT beliefs and attitudes CCT enrollment	Multivariate logistic regression
VanderWalde et al.	2022	Case-control study	Archival database	Alliance for Clinical Trials in Oncology SEER	Age Trial characteristics: disease site, trial phase, # trial modalities Enrollment disparity difference	Linear regression
Wagar et al.	2022	Case-control study	Archival database	ClinicalTrials.gov SEER (1992–2018)	Race/ethnicity Sex Age Cancer type Enrollment fraction	Enrollment fractions with odds ratios
Yekedūz et al.	2021	Case-control study	Archival database	FDA drug approvals (1/1/2006–6/30/2020) ClinicalTrials.gov SEER	Age Sex Race/ethnicity Comorbidity presence (including HBV, HBC, HIV) Organ dysfunction Brain metastases ECOG CCT reporting on the above variables CCTs reporting certain characteristics as exclusion criteria	FDA phase III CCTs/MEDLINE (1/1/2006–6/30/2020) SEER
Yonemori et al.	2010	Case-control study	Archival database	NDA trials (1999–2008) Ministry of Health, Labor, Welfare) SEER (2002–2006)	Median age of enrollees and proportion of those >65 by cancer site, drug, and application Age-specific incidence from Cancer Statistics in Japan (2013) * age-specific population (MHLW) to estimate age-specific new cases SEER for age-specific accrual information	Comparison of age median in US and Japanese populations to that of enrollees Comparison of proportion >65 in US and Japanese populations to that of enrollees
Zafar et al.	2011	Cohort study	Internal treatment center, archival database	KCI Phase I clinical trial service (1995–2005)	Via retrospective medical review: Demographics: age, gender, race Tumor type, PS, Tx status, enrollment status, Tx details, referring physician Three orthogonal groups: considered not enrolled (PC), enrolled but not treated (PE), treated (PT)	Fisher’s exact test
Zhao et al.	2024	Meta-analysis	Archival database	Clinicaltrials.gov (through 9/13/2022)	Difference in median age (CCT v. population) Age reporting Annual percent change	Joinpoint regression Mann–Whitney U and Kruskal–Wallis test
Zullig et al.	2016	Case-control study	Archival database	CTEP (1996–2009) NCCCR (1996–2009)	Incidence data: North Carolina Central Cancer Registry (NCCCR) Trial accrual data via CTEP Area Health Resource Files for certain demographic characteristics Accrual rate: #annual enrollment/# new cases, stratified by race, sex, county, and year	Preliminary chi-square tests Logistic regression
Zuniga et al.	2020	Case-control study	Archival database	ClinicalTrials.gov (Feb 2000–Feb 2019) SEER (2001–2015) American Joint Committee on Cancer (6e) TNM staging data (2004–2015)	Study representation proportion Identification of targets	One-sample proportion tests

*Note:* Abbreviations included in this table are utilized as follows, listed alphabetically: AA = African American; AAPI = Asian American or Pacific Islander; ACOSOG = American College of Surgeons Oncology Group; AI/AN = American Indian/Alaska Native; ANOVA = analysis of variances; API = Asian or Pacific Islander; B = Black; BMC = Boston Medical Center; CBTRUS = Central Brain Tumor Registry of the United States; CCC = comprehensive cancer center; CCT = cancer clinical trial; CCR = California Cancer Registry; CCSG = Cancer Center Support Grant; CDC = Centers for Disease Control and Prevention; CDUS = Clinical Data Update Service; CI = confidence interval; CINAHL = Cumulative Index of Nursing and Allied Health Literature; CSPOC = Cancer Surveillance Program of Orange County; CTED = Clinical Trials on Chronic Thromboembolic Disease; CTEP = Clinical Trial Evaluation Program; CTMS = Clinical Trials Management System; DF/HCC = Dana-Farber/Harvard Cancer Center; ECOG = Eastern Cooperative Oncology Group; EMPacT = Enhancing Minority Participation in Cancer Clinical Trials; EH/MR = electronic health/medical record; FDA = Food and Drug Administration; FFS = fee-for-service; GLOBOCAN = Global Cancer Observatory; GOG = Gynecologic Oncology Group; H = Hispanic; HS = high school; JH-SKCCC = Johns Hopkins Sidney Kimmel Comprehensive Cancer Center; ISS = International Staging System; KCI = Karmanos Cancer Institute; L = Latine; LACCSP = Los Angeles County Cancer Surveillance Program; MDCSS = Metropolitan Detroit Cancer Surveillance System; MDS = myelodysplastic syndromes; MHLW = Ministry of Health and Labor, Welfare; NCDB = National Cancer Database; NCI = National Cancer Institute; NDA = new drug application; NH = non-Hispanic; NIH = National Institutes of Health; O:E = observed:expected; NR = not reported; PDQ = Physician Data Query; PS = performance status; SEER = Surveillance, Epidemiology, and End Results; SE = standard error; SES = socioeconomic status; SWOG = Southwest Oncology Group; TCR = Thames Cancer Registry; TNM = Tumor Nodes Metastases; UCSF = University of California – San Francisco; W = White.

**Table 3.** Social, economic, and medical indicators of marginalization

Authors	Year	Age	Race/ethnicity	Sex	SGM status	SES	Ability & Comorbidities
Abbas et al.	2022	Among accruals: 65+: 32.1% Among population controls: 65+: 59.1%	Among accruals: NHW: 80.9% NHB: 7.6% AAPI: 3.3% H: 5.5% Other: 2.8% Among population controls: NHW: 77.5% NHB: 11.9% AAPI: 3.2% H: 5.6% Other: 1.7%	NR	NR	Among accruals: Income <$40k: 15.7% Private insurance: 57.5% ZIP HS edu<79%: 16.2% Among population controls: Income <$40k: 19.6% Private insurance: 34.3% ZIP HS edu<79%: 21.8%	NR
Abi Jaoude et al.	2020	NR	NR	NR	NR	NR	All trials: ECOG 0–1: 96.4% ECOG 2–4: 3.6%
Acoba, Sumida, and Berenberg	2022	NR	Of accruals: White: 35% Chinese: 6% Filipino: 16% Japanese: 27% Native Hawaiian: 16% Of population controls: White: 31% Chinese: 7% Filipino: 11% Japanese: 29% Native Hawaiian: 22%	NR	NR	NR	NR
Ajewole et al.	2021	NR	Of all participants: White: 71.5% Asian: 16.9% Black: 2.5% Hispanic: 2.3%	NR	NR	NR	NR
Al Hadidi et al.	2022	NR	2–5% (per study)	NR	NR	NR	NR
Aldrighetti et al.	2021	Used age-adjusted incidence rates	Of accruals: NHW: 82.3% B: 10.0% AAPI: 4.1% H: 3.4% AI/AN: 0.3%	NR	NR	NR	NR
Awad et al.	2020	Address via age-adjusted incidence	CCT participants (1995–2018) W: 79% B: 6% Other: 16%	Women	NR	Briefly address potential role of SES	NR
Baldini et al.	2022	Of participants: <70: 82.3% 70+: 17.7%	NR	Of <70 survey participants: F: 55.5 Of 70+ survey participants: F: 47.6%	NR	Of <70 survey participants: <HS: 60.4% FDI: −0.4 Of 70+ survey participants: <HS: 46.9% FDI: -0.3	NR
Baquet, Ellison, and Mishra	2009	Among accruals: 0–19: 13.8% 20–59: 48.3% 60+: 37.9%	Among accruals: (1999–2002, by sex) WM: 29.5% BM: 9.6% WF: 45.6% BF: 10.3% OM: 2.4% OF: 4.2%	Among accruals: F: 59.2%	NR	% of accrued patients among age-adjusted incidence within each category Lowest quartile material deprivation: 2.91% (F), 1.48% (M) Highest quartile material deprivation: 1.58% (F), 1.62% (M) Lowest quartile social class: 1.67% (F), 1.46% (M) Highest quartile social class: 3.15% (F), 1.85% (M) Insurance comparisons within accruals and incidence population % uninsured Accruals: 3.4% Population: 13.4% % Medicaid Accruals: 3.2% Population: 6.5% % Private Accruals: 65.4% Population: 77%	Briefly discuss potential role of comorbidity in compromising diverse representation
Behrendt, Hurria, Tumyan, Niland, and Mortimer	2014	Of total patients: *M* = 55.7	Of total patients: Other Caucasian: 42.2% African: 5.3% Asian: 16.3% Eastern European: 1.3% Latin American: 28.3% Middle Eastern: 6.5%	Women	NR	Of total patients: ZIP-code median income: <$45,000: 14.4% $45,500–$65,499: 37.4% $65,500–$85,499: 32.9% $85,000+: 15.3% Zip-code %racial/ethnicity-matched women 25+ without HS edu <5%: 20.9% 5–30%: 60.3% 30+%: 18.8%	NR, comment on lack of availability in limitations
Bero et al.	2021	NR	Race Of US CCT participants: W: 84.8% B: 11.8% Asian: 2.9% Other: 0.5% Of population: W: 72.2% B: 12.7% Asian: 5.6% Other: 9.5% Ethnicity Of US CCT participants: H: 9.8% Of population: H: 18.1%	Of US CCT participants: F: 41.5%	NR	NR	NR
Borad et al.	2020	Of CCT enrollees: Mean: 65.8 Average Median: 63.3 MM epidemiology: Mean: 71.5 Average Median: 71.5 MM epidemiology: Mean: 71.5 Average Median: 71.5	NR	NR	NR	NR	NR
Borno et al.	2019	Of accruals: 0–64: 70% 65+: 30%	Of accruals: NHW: 72% NHB: 4% Asian: 12% Hispanic: 10% Other: 2%	Of accruals: F: 46%	NR	Of accruals: Medicaid: 8% Medicare: 31% Private: 40% Other: 1% Missing: 20% Uninsured: 0%	NR
Brierley et al.	2020	Of non-accruals: Median: 69 (IQR: 61–76) Of accruals: Median: 68 (IQR: 61–73 years)	Of non-accruals: W: 87% B: 5.2% Asian: 1% Other: 6.9% Hispanic: 5.4% Of accruals: W: 88% B: 4.2% Asian: 1.6% Other: 6.2% Hispanic: 3.8%	Of non-accruals: F: 39% Of accruals: F: 29%	NR	Of non-accruals: <$48,138: 26.6% $48,138–$63,083: 25.4% $63,083–$90,412: 24.4% $90,412+: 23.5% Of accruals: <$48,138: 19.6% $48,138–$63,083: 23.5% $63,083–$90,412: 27.4% $90,412+: 29.5%	Report on comorbidity, functional status, and underrepresented disease as focus
Bruno, Li, and Hess	2024	Mean: 59.5	% Black (total): 25.2%	Total: F: 47.3%	NR	Applied to Medicaid-exclusive population	Report on disease characteristics
Bruno et al.	2022	Total means: NSCLC: 68.9 NS-NSCLC: 68.3 CRC: 63.1 Breast: 63.8	% of White participants (relative to all White patients):: NSCLC: 3.9% NS-NSCLC: 3.9% CRC: 2.9% Breast: 5.8% % of Black participants (relative to all Black patients): NSCLC: 1.9% NS-NSCLC: 1.2% CRC: 2.9% Breast: 4.4%	Total % F: NSCLC: 48.1% NS-NSCLC: 52.6% CRC: 43.5% Breast: 99.0%	NR	Reported insurance status across patients, clinic practice volume, and practice type stratified by diagnostic site and dichotomized race	Reported staging and ECOG stratified by diagnostic site and dichotomized race
Canoui-Poitrine et al.	2019	% group CCT invited: 65–69: 39% 70–74: 30% 75–79: 24% 80+: 7% Of SAGE population: 65–69: 27% 70–74: 23% 75–79: 23% 80+: 27% Of SAGE population: 65–69: 27% 70–74: 23% 75–79: 23% 80+: 27%	NR	% group CCT invited: M: 55% Of SAGE population: M: 56% Of SAGE population: M: 56%	NR	Of SAGE population: Higher education: 34%	% group CCT invited: Comorbidity: 67% Performance status 2+: 8% Of SAGE population: Comorbidity: 73% Performance status 3–4: 4% Of SAGE population: Comorbidity: 73% Performance status 3–4: 4%
Casey et al.	2023	Of RCT participants: Mean: 57.3	Of RCT participants: W: 83.2% AAPI: 6.3% B: 3.2% H: 6%	Of RCT participants: F: 40.5%	NR	Commented on geographical distribution of RCTs and intersection of county-level insurance coverage with race	Briefly address impact of staging and comorbidities
Costa, Hari, and Kumar	2016	Of non-accruals: Median = 69 Of accruals: Median = 61	Expected: %minority accruals: 36.7% Observed: %minority accruals: 19.1%	Expected male% accruals: 58.4% Observed male% accruals: 56.9%	NR	NR	Reported on higher enrollment of lower risk patients by stage I: 37.1% II: 39% III: 24.8%
Choradia et al.	2024	Of participants: 65+: 22.9%	Of participants: W: 76.1% B: 12.0 AAPI: 4.6% AI/AN: 0.3% H: 7.1%	Of participants: F: 41.7%	NR	NR	NR
Craig, Gilbery, Herndon, Vogel, and Quinn	2010	Of non-accruals: Median (IQR): 73 (69–78) Of accruals: Median (IQR): 72 (68–76)	Of non-accruals: White: 80% Black: 9% Hispanic: 4% Other: 6% Of accruals: White: 85% Black: 6% Hispanic: 4% Other: 5%	Men	NR	Of non-accruals: Median income (IQR): $46,273 ($35,351–$61,363) Of accruals: Median income (IQR): $51,656 ($38,763–$69,754)	Of non-accruals: Comorbidity index 0: 75% Of accruals: Comorbidity index 0: 78%
Diehl et al.	2011	NR	Range of proportions, of accruals: early-stage breast: AA 6.4–14.0% HA 2.7–4.0% regionally advanced breast: AA 14.0–15.2% HA 4.2–4.8% non-metastatic lung: AA 8.0–11.0% HA 2.7–2.3% metastatic lung: AA 11.3% HA 2.6%	NR	NR	Briefly report on SES in introduction, scarcely address in discussion	Briefly report on early-stage eligibility criteria prohibiting diverse representation
Dressler et al.	2015	Of accruals: Age median (range): 58.3 (18.8–93.5)	Of accruals: White: 83.0% AA: 11.1% Asian: 2.5% Other: 1.0% Unknown: 2.4% White: 85.1% non-White: 14.9%	Of accruals: F: 59.3%	NR	NR	NR
Du, Gadgeel, and Simon	2006	Of non-accruals: 70+: 24% Of accruals: 70+: 10%	Of non-accruals: AA: 45% non-AA: 55% Of accruals: AA: 25% non-AA: 75%	Of non-accruals: F: 43% Of accruals: F: 32%	NR	Of non-accruals: Low: 52% Medium: 28% High: 21% noncommercial insurance: 63% Of accruals: Low: 37% Medium: 30% High: 33% non-commercial insurance: 45%	Of non-accruals: PS = 0: 31% Heart disease: 18% Diabetes: 13% COPD: 16% Comorbidities >0: 39% Of accruals: PS = 0: 36% Heart disease: 16% Diabetes: 9% COPD: 13% Comorbidities >0: 31%
Dudipala et al.	2023	Of total cohort assessed: M: 70	Of total cohort assessed: B: 35.1% W: 47.5% H: 9.9%	Of total cohort assessed: F: 47.5%	NR	Of total patients: HS edu or <: 77.9% <$84k median household income: 70.6%	Accounted for staging/subtype (25%) comorbidities/low functional status (17.6%) as potential limiting factor for enrollment among CCT discussed subsample
Duma et al.	2018	Of current trial participants: 65+: 36.0% 2013 SEER: 65+: 60.0%	Of current trial participants: NHW: 83.4% AA: 6.0% H: 2.6% AAPI: 5.3% AI/AN: 0.3% Other: 2.4% 2013 SEER: NHW: 79.0% AA: 10.0% H: 7.0% AAPI: 3.3% AI/AN: 0.3% Other: NR	Of current trial participants: F: 41.0% 2013 SEER: F: 50.0%	NR	NR	NR
Earl et al.	2023	NR	Of enrollees: W: 93.2% B: 0.9% Asian: 1.2% NH/PI: 0.4% AI/AN: 0.0% O: 2.8%	Of enrollees: F: 42.6%	NR	Sex, race, and ethnicity outcomes stratified by county classification County income and edu utilized as secondary analysis predictors	NR
Elshami et al.	2022	Of total patients: 70+: 41.0%	Of total patients: NHW: 70.0% NHB: 12.4% H: 7.8% O: 9.8%	Of total patients: F: 41.5%	NR	Of total patients: <$53,353: 40.0% “Less educated:” 46.5% Private insurance: 31.5% Distance 11.6 mi+: 47.4%	Of total patients: Charlson-Deyo score 2+: 14.7% Stage 4: 40.1%
Eskander et al.	2022	Of enrollees: M: 64.0 Of non-enrollees: M: 69.0	Of enrollees: W: 90.1% NW: 9.9% Of non-enrollees: W: 83.2% NW: 16.8%	Of enrollees: %F: 46.0% Of non-enrollees: %F: 48.9%	NR	Of enrollees: Private insurance: 49.4% Median income <$38k: 11.8% <HS 21%+: 9.3% Non-metropolitan: 14.5% Of non-enrollees: Private insurance: 30.9% Median income <$38k: 17.7% <HS 21%+: 16.9% Non-metropolitan: 15.5%	Of enrollees: Charlson-Deyo 1+: 22.5% Stage 4: 65.8% Of non-enrollees: Charlson-Deyo 1+: 34.7% Stage 4: 52.0%
Fakhry et al.	2023	NR	Of cumulative enrollees in all studies: W: 83.7% B: 5.1% AI/AN: 0.0% Asian: 0.14% NH/PI: 0.0% Multiracial: 0.0% H: 2.2%	Briefly address sex/gender reporting	NR	Addresses intersectionality between racial/ethnic representation and low socioeconomic strain	NR
Fayanju et al.	2019	Non-accruals: <40: 5% 40–64: 67.1% 65+: 27.9% Accruals: <40: 5.6% 40–64: 56.3% 65+: 38.1%%	Non-accruals: NHW: 73.7% NHB: 10.7% API: 3% Native American: 0.3% Hispanic: 5% Other: 6.4% Accruals: NHW: 83.5% NHB: 7.3% API: 2.4% Native American: 0.2% Hispanic: 4% Other: 2.6%	Women	NR	Non-accruals: <$38,000: 15.4% $38,000–47,999: 21.1% $48,000–62,999: 26.2% $63,000+: 35.6% >93% of area HS grad: 27.4% Accruals: <$38,000: 12.9% $38,000–47,999: 19.8% $48,000–62,999: 24.8% $63,000+: 33% >93% of area HS grad: 32.5% Explicitly reported on race/ethnicity*SES intersection	Briefly discuss comorbidities and effects of ECOG performance status on age and racial underrepresentation
Freudenburg et al.	2022	Reported per study included	Of study participants: W: 81–98% AA: 2–8% H: 2–5%	Reported per study included	NR	NR	NR
Gopishetty, Kota, and Guddati	2020	Of accruals: Colon %65+: 28.8% Lung %65+: 38.8% Breast %65+: 14.7% DLBCL %65+: 39.2% AML %65+: 29.0% ALL %65+: 9.6%	Of accruals: Colon Asian: 21.2% AA: 2.6% W: 74.3% Other: 2.0% Lung Asian: 26.1% AA: 30.6% W: 39.7% Other: 3.7% Breast Asian: 17.4% AA: 3.6% W: 73.8% Other: 5.2% DLBCL Asian: 16.6% AA: 1.5% W: 77.7% Other: 4.2% AML Asian: 2.3% AA: 2.3% W: 92.9% Other: 2.5% ALL Asian: 5.9% AA: 6.7% W: 77.5% Other: 9.9%	NR	NR	NR	Contextualize age-related disparities in comorbidity risk and ineligibility
Gordis et al.	2022	Participants: M: 59 years NCDB: M: 58.4 years	Participants: W: 88.2% AA: 4.8% H: 1.8% AAPI: 0.3% Other: 2.5% NCDB: W: 67.7%	Participants: F: 11.8% NCDB: F: 32.1% NCDB: F: 32.1%	NR	NCDB only: High SES: 65.2%	Participants: No smoking Hx: 50% No alcohol use: 28.7% Primary tongue site: 41.4% NCDB: Primary tongue site: 65.2% NCDB: Primary tongue site: 65.2%
Grant et al.	2020	Briefly mention possible effects of age in disparities	Report explicitly on racial/ethnic representativeness of CCTs	NR	NR	Briefly mention possible effects of SES factors in disparities	NR
Green et al.	2022	CCT participants: 75+: 85+: Non-participants: 75+: 85+:	CCT participants: Asian: 1.5% B: 5.8% W: 86.7% Oth: 6.0% Non-participants: Asian: 1.5% B: 8.0% W: 86.0% Oth: 4.5%	CCT participants: M: 55.3% Non-participants: M: 49.5%	NR	CCT participants: Median income $60,430+: 57.0% Metro: 87.2% Non-participants: Median income $60,430+: 47.4% Metro: 81.7%	CCT participants: Charlson score 2+: 13.1% Non-participants: Charlson score 2+: 26.1%
Grette et al.	2021	Accounted for age-adjustment in comparisons	Of CCT participants: W: 70% B: 5% Asian: 20% Other: 6%	Primarily AFAB (i.e., breast, GYN)	NR	NR	NR
Gross, Filardo, Mayne, and Krumholz	2005	Restricted sample to 65+ Of accruals: 65–69: 43.4% 70–74: 29.2% 75–79: 21.0% 80+: 6.4% Of non-accruals: 65–69: 25.4% 70–74: 26.5% 75–79: 23.0% 80+: 25.1%	Of accruals: White: 86.7% AA: 4.9% Asian: 5.4% Hispanic: 3.0% Of non-accruals: White: 88.3% AA: 7.0% Asian: 3.0% Hispanic: 1.7%	Women	NR	Of accruals: %Medicaid: 2% 0.13%+ below poverty level: 20.9% % unemployment 5.6+: 18.7% Of non-accruals: %Medicaid: 10% 0.13%+ below poverty level: 24.9% % unemployment 5.6+: 25.1%	Speculate on relationships between SES and later staging
Guerrero et al.	2018	NR	NR: 67.0% W: 25.9% Asian: 5.0% AA: 1.1% H: 0.2% Other: 0.9%	NR	NR	NR	NR
Hantel et al.	2024	Total: Median: 67	Total: NHW: 85.9% NHB: 4.3% NHA: 3.7% HW: 4.5% Oth: 1.3%	Total: F: 45%	NR	Total: sYI: 6/10 Distance: 50 km Private insurance: 30.6%	Briefly comment on limited availability of such data and potential role
Hantel et al.	2022	Of enrollees: 60–79: 38.8% 80+: 2.5%	Of enrollees: NHW: 81.7% NHB: 7.5% NH-NA: 0.88% NH-Asian: 2/44% H: 5.33% Oth: 2.17%	Of enrollees: F: 42.4%	NR	Of enrollees: Area deprivation index 76–100%ile: 18.10% Urban: 76.4% CCC: 62.5%	NR
Hanvey et al.	2022	Of total: 60+: 56.2%	Of total: Non-White: 15.9% Hispanic: 5.1% POC: 20.3%	All AFAB	NR	Examined as longitudinal attrition predictor	Depression, anxiety, pain, and sleep examined as longitudinal attrition predictor
Hennessy et al.	2022	Median age: 62 y.o. Age restriction: 32% Median age restriction: 75	NR	NR	NR	NR	NR
Hori et al.	2007	Of all Japanese cancer population: Median(range) = 69 (54–75) %65+: 64% Of Japanese CCT accruals (68 trials): Median difference compared to population: 7 (−16–33) %trials median age < population: 88.2% Report explicitly on underrepresentation of older (65+) cancer patients	Japanese nationality (no further specification)	NR	NR	NR	Contextualized findings within comorbidity, functional status, and eligibility criteria
Housri et al.	2015	Of accruals: <60: 60% 65+: 40% Of non-accruals: <60: 55.3% 65+: 44.7%	Of accruals: Dichotomized W: 74.6% NW: 25.4% Full categories: NHW: 74.6% Black: 13.1% Asian: 6.9% Hispanic: 5.4% Of non-accruals: Dichotomized W: 59.8% NW: 40.2% Full categories: NHW: 59.8% Black: 15.2% Asian: 12.9% Hispanic: 12.1%	Women	NR	NR	Staging Of accruals: Tis = 22.3% T1 = 69.2% T2 = 8.5% Of non-accruals: Tis = 18.2% T1 = 59.8% T2 = 22%
Huang, Ezenwa, Wilkie, and Judge	2013	Of total pain referrals: *M* = 53.6 Of total symptom referrals: *M* = 52.9	Of total referrals: NHW: 79% Minority: 13% Unknown: 8%	Of total referrals: M: 41% F: 59%	NR	NR	NR
Hue et al.	2022	Of CCT enrollees: Stage I–III: 64 Stage IV mean: 63 Of non-enrollees: Stage I–III: 69 Stage IV mean: 68	Of CCT enrollees: Stage I–III: NHW: 86.3% NHB: 5.7% Oth: 8.0% Stage IV: NHW: 85.9% NHB: 4.8% Oth: 9.3% Of non-enrollees: Stage I–III: NHW: 75.9% NHB: 10.9% Oth: 13.2% Stage IV: NHW: 73.9% NHB: 12.1% Oth:14.0%	Of CCT enrollees: Stage I–III: F: 49.5% Stage IV: F: 44.5% Of non-enrollees: Stage III: F: 50.4 Stage IV: F: 46.9%	NR	Of CCT enrollees: Stage I–III: Median ZIP income <$40,227: 13.4% ZIP w/o HS Edu 17.6%+: 12.9% Private insurance: 47.5% Stage IV: Median ZIP income <$40,227: 11.4% ZIP w/o HS edu 17.6%+: 11.6% Private insurance: 51.7% Of non-enrollees: Stage I–III: Median ZIP income <$40,227: 18.6% ZIP w/o HS Edu 17.6%+: 20.9% Private insurance: 31.3% Stage IV: Median ZIP income <$40,227: 19.1% ZIP w/o HS Edu 17.6%+: 20.6% Private insurance: 32.5%	Of CCT enrollees: Stage III: Charlson-Deyo 3+: 1.2% Stage IV: Charlson-Deyo 3+: 0.7% Of non-enrollees: Stage III: Charlson-Deyo 3+: 2.9% Stage IV: Charlson-Deyo 3+: 3.5%
Jan et al.	2022	Of all CCT participants: 65+: 46.7%	Of all CCT participants: W: 44.3% B: 3.6% AAPI: 47.4% Unk: 4.4%	Of all CCT participants:	NR	NR	NR
Javid et al.	2012	Among eligible respondents: %65+ trial available: Yes: 27% No: 30% %65+ trial eligible: Yes: 24% No: 37% %65+ trial enrolled: Yes: 21% No: 26% %65+ trial eligible: Yes: 24% No: 37%	NR	AFAB-exclusive	NR	% Distance >50 mi, trial enrolled: Yes: 23% No: 34%	Addressed at item level regarding reasons for ineligibility and intersection with age (dichotomized 65+)
Javier-DesLoges et al.	2022	Of CCT participants: 65+: 33.8% US rate: 65+: 44.1%	Of CCT participants: NHW: 81.3% B: 8.7% H: 4.8% AAPI: 2.8% NA: 0.3% Oth: 2.0% US rate: NHW: 78.5% B: 11.6% H: 5.9% AAPI: 2.6% NA: 0.5% Oth: 0.9%	Of CCT participants: F: 71.7% US rate: F: 49.2%	NR	NR	NR
Jayakrishnan et al.	2021	Of CCT participants: M: 61	Race reporting only: 85.4%	NR	NR	Briefly mention potential role in explaining findings	Briefly mention potential role in explaining findings
Kaanders et al.	2022	Age restriction: 42% CCT participants: Median: 57 years Clinical population: 64 years	NR	NR	NR	NR	>70 Karnofsky restriction: 18% CCT participants: >70%: 0–1 PS or 90–100 Karnofsky WHO/ECOG/ Zubrod restriction 0–1: 21% CCT participants:
Kanapuru et al.	2023	Of screened patients: 65–75: 41% 75+: 19%	Of screened patients: W: 83% Asian: 7% B: 4% Oth: 2% H: 4%	Of screened patients: F: 45%	NR	NR	Briefly address potential role of comorbidity
Kanarek et al.	2010	Of non-accruals: Baltimore: <20: 3.1% 20–64: 58.8% 65+: 38.1% Non-Baltimore: <20: 2.8% 20–64: 64.4% 65+: 32.8% Non-catchment area: <20: 2.2% 20–64: 68.8% 65+: 29.0% Of accruals: Therapeutic: <20: 9.9% 20–64: 61.7% 65+: 25.5% Non-therapeutic: <20: 9.7% 20–64: 66.9% 65+: 20.1%	Of non-accruals: Baltimore: W: 43.0% B: 55.4% O: 1.6% Non-Baltimore: W: 85% B: 11.0% O: 4.0% Non-catchment area: W: 91.6% B: 5.4% O: 3.0% Of accruals: Therapeutic: W: 85.4% B: 10.9% O: 3.6% Non-therapeutic: W: 83.1% B: 13.8% O: 2.7%	Of non-accruals: Baltimore: M: 49.8% F: 50.2% Non-Baltimore: M: 58.5% F: 41.5% Non-catchment area: M: 72.9% F: 27.1% Of accruals: Therapeutic: M: 57.5% F: 42.5% Non-therapeutic: M: 54.7% F: 45.3%		County poverty quartiles: Of non-accruals: Baltimore: Least poor: 0% 2: 0% 3: 0% Poorest: 100% Non-Baltimore: Least poor: 87.6% 2: 8.9% 3: 0.5% Poorest: 3.0% Non-catchment area: Least poor: 30.2% 2: 23.6% 3: 20.2% Poorest: 9.6% Of accruals: Therapeutic: Least poor: 69.4% 2: 10.9% 3: 4.7% Poorest: 12.6% Non-therapeutic: Least poor: 68.9% 2: 9.1% 3: 4.7% Poorest: 14.5%	NR
Keegan et al.	2023	NR	Race reporting: 73.4% studies reported race/ethnicity	NR	NR	NR	NR
Khadraoui et al.	2023	Of CCT enrollees: M: 60.4 Of non-enrollees: M: 62.9	Of CCT enrollees: W: 85.8% B: 7.1% H: 3.8% Asian: 2.2% NH/PI: 0.2% AI/AN: 0.6% Oth: 9.7% Of non-enrollees: W: 78.7% B: 10.0% H: 6.8% Asian: 3.3% NH/PI: 0.3% AI/AN: 0.3% Oth: 11.3%	All AFAB	NR	Of CCT enrollees: Private insurance: 58.2% Median income <$46,277: 12.4% %w/o HS edu 15.3%+: 12.2% Rural: 1.6% Of non-enrollees: Private insurance: 45.8% Median income <$46,277: 16.9% %w/o HS edu 15.3%+: 21.5% Rural: 1.6%	Of CCT enrollees: Charlson-Deyo 2+: 2.7% Stage IV: 26.6% Of non-enrollees: Charlson-Deyo 2+: 5.7% Stage IV: 12.4%
Kilic et al.	2023	Of age-reporting CCTs (avg): 65+: 51%	Of race-reporting CCTs (avg): NHW: 82% NHB: 9% NHAPI: 4% NHAIAN: 0.3% NHUR: 3 H: 2%	Of sex-reporting CCTs (avg): F: 44%	NR	Briefly discuss potential role of SES	NR
Ko et al.	2015	Of total screens: *M* = 61	Of total screens: NHW: 44% NHB: 40% Hispanic: 9% Asian: 3% Other: 4%	Of total screens: M: 39% F: 61%	NR	Of total screens: Insurance Public: 66% Private: 24% Uninsured: 10% Education HS: 26% <HS: 21% >HS: 13% Employment: Employed: 21% Unemployed: 31% Retired: 35% Disabled: 12%	Accounted for ability and comorbidities as reasons for ineligibility and non-enrollment
Kwak et al.	2023	Of CCT enrollees: M: 63.7 Of non-enrollees: M: 68.4	Of CCT enrollees: NHW: 81.9% NHB: 7.2% H: 2.2% Oth: 8.8% Of non-enrollees: NHW: 78.5% NHB: 10.3% H: 2.9% Oth: 8.3%	Of CCT enrollees: F: 53.8% Of non-enrollees: F: 47.8%	NR	Of CCT enrollees: Private insurance: 42.2% Distance traveled: 55.8 mi. Lowest SES sector: 6.6% Of non-enrollees: Private insurance: 26.2% Distance traveled: 27.2 mi. Lowest SES sector: 11.0%	Of CCT enrollees: Charlson Deyo 3+: 2.9% Stage IV: 67.3% Of non-enrollees: Charlson Deyo 3+: 4.9% Stage IV: 40.2%
Ladbury et al.	2022	Mean age difference (participants vs. SEER): −2.29 years	EIR (participants vs. SEER) W: 1.06 B: 0.86 Asian: 0.51 AI/AN: 0.74 H: 0.89 H: 0.89	NR	NR	NR	NR
Langford et al.	2014	Of all patients: M: 62 65+: 43%	% enrollment rate within racial/ethnic group: NHW: 20% NHB: 18% Hispanic: 22% Asian: 10% Other: 14% Proportion of all patients: NHW: 78% NHB: 13% Hispanic: 4% Asian: 4% Other: 1%	F: 68%	NR	NR	Addressed demographic characteristics as predictors of comorbidity
Lythgoe, Savage, and Prasad	2021	NR	Of race-reporting CCTs: W; 76.3% B: 2.9% Asian: 7.9% AI/AN: 0.5% Oth: 1.8% Unknown/missing: 10.5%	NR	NR	NR	NR
Mishkin, Minasian, Kohn, Noone, and Temkin	2016	Of accruals: 75–84: 7.1% 85+: 0.4% Population estimates: 75–84: 18.5% 85+: 10.4%%	Of accruals: White: 87.8% Black: 8.3% AI/AN: 0.9% API: 3.0% Hispanic: 5.9% Non-Hispanic: 94.1% Population estimates: White: 81.7% Black: 13.4% AI/AN: 0.6% API: 4.3% Hispanic: 14.7% Non-Hispanic: 85.3%	Women	NR	Of accruals: Private (ovarian): 85.8% Medicaid: 5.5% Uninsured (cervical): 15.8% Population estimates: Private (ovarian): 76.1% Medicaid: 13.9% Uninsured (cervical): 8.9%	Comment on intersection between age, race/ethnicity, SES, and ability
Moloney and Shiely	2022	Addressed disproportionate impact of eligibility criteria (i.e., ECOG, complications) on older adult underrepresentation	Addressed disproportionate impact of eligibility criteria (i.e., differences in organ functioning, comorbidities) on underrepresentation of Black and Hispanic participation	Primary AFAB focus	Addressed disproportionate impact of eligibility criteria (i.e., blood-borne virus and associated treatment) on LGBTQ + underrepresentation	Addressed disproportionate impact of eligibility criteria (i.e., blood-borne virus and associated treatment, differences in organ functioning, comorbidities) on underrepresentation of individuals with lower SES	Addressed disproportionate impact of eligibility criteria on individuals experiencing physical, cognitive, or psychiatric comorbidity; or on individuals experiencing treatment complications, metastases, or poorer functional status
Murthy, Krumholz, and Gross	2004	Of accruals: 30–64: 68% 65–74: 23.7% 70+: 8.3% Population estimates: 30–64: 37.5% 65–74: 31.4% 75+: 31.2%	CCT enrollees White: 85.6% Black: 9.2% API: 1.9% AI/AN: 0.3% Hispanic: 3.1% Population estimates: White: 83.1% Black: 10.9% API: 2% AI/AN: 0.2% Hispanic: 3.8%	Of accruals: M: 32.1% F: 67.9% Population estimates: M: 51% F: 49%	NR	Report briefly on potential SES intersection with race/ethnicity in compromising participation)	Briefly alluded to potential comorbidity intersection with age and race/ethnicity in compromising participation
Newman et al.	2004	Of ACOSOG accruals: <65: 56% 65+: 44% Population estimates: <65: 42.8% 65+: 57.2%	Of all accruals: AA: 10.5% Hispanic: <1% Population estimates: AA: 9.4% Hispanic: 3.4%	Elaborate in discussion on interactions between race, ethnicity, and sex	NR	Elaborate in discussion on interactions between race, ethnicity, and SES	Directly account for more advanced staging among minority patients at initial presentation; refers to eligibility limitations in discussion for older adults
Noor et al.	2013	Of referrals: <67: 68.4% 67+: 31.6% Of comparators: <67: 44.9% 67+: 55.1%	Of referrals: W: 74.2% NW: 13.7% Unspecified: 12.1% Of comparators: NR	Of referrals: M: 54.7% F: 45.3% Of comparators: M: 51.9% F: 41.8%	NR	Of referrals: IMD 1: 15.8% IMD 2: 14.7% IMD 3: 20.7% IMD 4: 27% IMD 5: 21.9% Of comparators: IMD 1: 13% IMD 2: 14.2% IMD 3: 16.3% IMD 4: 29% IMD 5: 27.4%	Allude briefly to intersection of age, SES, and ability via discussion of comorbidities
Osann et al.	2011	Of accruals: NH *M* = 48.1 H *M* = 50.8	Of accruals: NH: 60% H: 40% 70% Hispanic enrollees: speak Spanish at home	Women	NR	Of accruals: Education (College+): NH: 80% H: 25% Income ($25k+): NH: 83.3% H: 37.5%	NR
Owens-Walton et al.	2022	Briefly addresses intersecting role of age in underpinning CCT disparities	Primary focus of representativeness (proportions NR, only relative representation)	NR	NR	Briefly addresses intersecting role of SES in underpinning CCT disparities	Briefly addresses intersecting role of comorbidities in underpinning CCT disparities
Palmer et al.	2021	% participating in any cancer research 65+: 21.9% Exclusion: 75+	% participating in any cancer research African American: 47.6% Asian American: 16.7% Latino: 17.0% White: 26.2%	AMAB only	NR	% participating in any cancer research HS or less: 18.7% Private insurance: 27.8% Low health literacy: 15.5%	Health status <very good: 24.1% Comorbidity 2+: 29.2% Exclusion: no physical, cognitive, mental disability
Pang et al.	2016	Of accruals: <70: 74.7% 70+: 25.3%	Of accruals: White: 87.4% Black: 7.7% AI/AN: 1.0% API: 1.3% Hispanic: 1.7% Non-Hispanic: 92.8%	Of accruals: M: 59.1% F: 40.1%	NR	Report partially on intersection between minorities, SES indicators, and access to clinic	NR
Patel et al.	2023	Of GI total: 65–74: 30% 75+: 18% Of HN total: 65–74: 19% 75+: 12%	Of GI total: W: 87% B: 7% Asian: 2% Oth: 2% Missing/unk: 1% Of HN total: W: 92% B: 3% Asian: 3% Oth: 2% Missing/unk: 1%	Of GI total: F: 40% Of HN total: F: 46%	NR	Of GI total: Not working: 19% Private insurance: 35% Of HN total: Not working: 14% Private insurance: 42%	Of GI total: <5 CCI: 29% Stage IV: 27% Of HN total: <5 CCI: 63% Stage IV: 40%
Patel et al.	2020	Of total sample: 50 and younger: 24% 51–65: 46% 65+: 30%	Of total sample: White: 56% Black: 18% Latina: 18% Asian: 9% High acculturation: 85%	Women	NR	Of total sample: Education HS or less: 29% Some college or technical: 32% College+: 39% Income <$40,000: 37% $40,00: 63% Insurance None: 1% Medicaid: 14% Medicare: 29% Other public: 1% Private: 55% Employment Unemployed: 61%	Of total sample: Comorbidity 0: 71% 1+: 29%
Patki et al.	2023	NR	Of CCT participants: W: 82.6% B: 9.8% Asian: 5.7% (greatest underrepresentation) H: 7.9%	AMAB only	NR	# reporting SES: 1 # reporting edu attainment: 3 Comment on lack of available data for reporting	Briefly comment on intersection between race, ethnicity, SES, and ineligibility
Perni, Moy, and Nipp	2021	Phase I: Median: 60 Phase II–III: Median: 61	Phase I: W: 93% B: 2% Asian: 6% Phase II–III: W: 93% B: 4% Asian: 3%	Phase I: F: 57% Phase II–III: F: 44%	NR	Phase I: Median income <$50k: 14% Distance <50: 57% Private insurance: 67% Phase II–III: Median income <$50k: 16% Distance <50: 72% Private insurance: 69%	Phase I: Metastatic: 79% Phase II–III: Metastatic: 59%
Pittel et al.	2023	Of patient total: 65–74: 32.7% 75 + 26.8%	% of group participating in CCTs: W: 7.2% B: 4.4% L: 4.2% Of total: W: 78.4% B: 13.7% L: 7.9%	Of total patients: F: 57.3%	NR	NR	Of total patients: ECOG 2+: 15.2%
Pirl et al.	2018	Note reporting across studies	Among race/ethnicity-reporting trials: W: 73.2% AA: 5.7% Asian: 9.9% Hispanic/Latine: 8.8%	Note reporting across studies	NR	Note reported SES variables for each study	NR
Ramamoorthy et al.	2018	Among CCT participants: 2008–2013: 65+: 41% 2014–2017: 65+: 39% 2014–2017: 65+: 39%	Among CCT participants: 2008–2013: W: 80% Asian: 12% B: 4% Hispanic: 4% Outside US: 74% 2014–2017: W: 71% Asian: 22% B: 1%	Among CCT participants: 2008–2013: F: 44% 2014–2017: F: 52%	NR	NR	NR
Riaz et al.	2023	Of CCT participants: 65+: 71.1%	Of CCT participants: B/AA: 10.8% AAPI: 1.5% W: 78.5% H: 4.4%	AMAB only	NR	NR	NR
Reihl et al.	2022	Age-adjusted comparison rates (cohort age NR)	Of CCT participants: W: 91.7% Asian: 1.5% B: 2.6% H: 1.7%	Of CCT participants: F: 37.5%	NR	NR	NR
Saphner et al.	2021	CCT enrollees: 65+:38.3%	CCT enrollees: W: 90.4% B: 6.6% NH Other: 1% H: 1.9%	CCT enrollees: F: 62.2%	NR	CCT enrollees: Median income: 0.25/1.00 Below PL: 6.3% Owner values: 0.18/1.00 Unemployed: 2.8% College: 27.2% <HS: 4.2% Crowding: 0	NR
Sawaf et al.	2023	Primarily addressed underrepresentation of older participants per trial	Primarily addressed relative underrepresentation of Black and Hispanic patients per trial Described significant underreporting of Asian, NH/PI, and AI/AN races	Primarily addressed underrepresentation of females per trial	NR	Address lack of CCT reporting SES, education, and rurality	Address lack of CCT reporting on comorbidity scores, limited ECOG, BMI, and smoking reporting
Scalici et al.	2015	Apply age-adjusted rates	Of accruals: White: 83% Black: 8% Other: 9%	NR	NR	NR	NR
Sedrak et al.	2022	Offered CCT: 50–69 y.o.: 74% 70+: 26% Enrolled in CCT: 50–69 y.o.: 68% 70+: 85% Enrolled in CCT: 50–69 y.o.: 68% 70+: 85%	Ethnicity (%Hispanic) 50–69 y.o.: 6% 70+ y.o.: 3%	F: 50–69 y.o.: 80% 70+ y.o.: 64%	NR	Income 50–69 y.o. <$50K: 34% 70+ y.o. <$50K: 47% Education 50–69 y.o. <HS: 6% 70+ y.o. <HS: 9% Rurality: 50–69 y.o. rural site: 21% 70+ y.o. rural site: 24% Education 50–69 y.o. <HS: 6% 70+ y.o. <HS: 9% Rurality: 50–69 y.o. rural site: 21% 70+ y.o. rural site: 24%	# Comorbidities 50–69 y.o., 2+: 301% 70+ y.o., 2+: 51%
Shinder et al.	2023	CCT participants: M: 56.4 Matched controls: M: 63.5	CCT participants: W: 90.3% B: 4.3% Oth: 3.7% Matched controls: W: 86.0% B: 10.3% Oth: 2.9%	CCT participants: F: 29.1% Matched controls: F: 37.2%	CCT participants: <$38k median income: 13.2% W/o HS edu 21%+: 11.2% Private insurance: 67.3% Matched controls: <$38k median income: 19.0% W/o HS edu 21%+: 17.9% Private insurance: 41.0%	CCT participants: Distance: 61.1 mi Matched controls: Distance: 32.9 mi	CCT participants: Stage IV: 20.7% Charlson-Deyo = 0: 81.6% Matched controls: Stage IV: 20.7% Charlson-Deyo = 0: 69.8%
Steventon et al.	2024	NR	% of CCT enrollees: AI/AN: 0.1% East Asian: 9.1% Asian (Oth, NOS): 0.5% B/AA: 3.7% Hispanic/Latino: 0.6% H/Unk/Unsp: 0.1% NH/PI: 0.1% Oth/unk: 6.1% Caucasian: 79.8% % of CCT enrollees by continent: North America: 80.1% (US: 78.1% total) Europe: 13.0% East Asia: 3.4% Middle East: 1.3% South American: 1.3% Australasia: 0.7%	NR	NR	NR	Briefly address potential contributing role of comorbidity
Stewart, Bertoni, Staten, Levine, and Gross	2007	Of accruals: 21–44: 16.53% 45–54: 28.23% 55–64: 28.08% 65–74: 20.61% 75+: 6.55% Population estimates: 21–44: 4.91% 45–54: 11.82% 55–64: 20.84% 65–74: 30.78% 75+: 31.64%	Of accruals: NHW: 86.57% Hispanic: 3.4% AA: 7.92% API: 1.86% AI/AN: 0.25% Population estimates: NHW: 82.15% Hispanic: 4.24% AA: 11.23% API: 2.16% AI/AN: 0.22%	Of accruals: M: 16.05% F: 83.95% Population estimates: M: 48.97% F: 51.03%	NR	Comment on intersection between minority status and SES	Comment on intersection between minority status and disqualifying cardiovascular comorbidities
Talarico, Chen, and Pazdur	2004	Of participants: 65+: 36% 70+: 20% 75+: 9% Of SEER: 65+: 60% 70+: 46% 75+: 31%	Reported “no imbalance by […] ethnicity”	Reported “no imbalance by sex”	NR	NR	NR
Tharakan, Zhong, and Galsky	2021	NR	% CCT enrollees overall: Black: 2.5% % CCT enrollees overall w/ location data: Black: 3.2%	NR	NR	Briefly address role of national SES	NR
Unger et al.	2020	NR	Pharmaceutical company: B: 2.9% SWOG: B: 9.0% SEER: B: 12.1% SWOG:	NR	NR	NR	NR
Unger, Gralow, Albain, Ramsey, and Hershman	2016	<65: 71% 65+: 29%	AA: 7% All other: 93%	M: 16% F: 84%	NR	Income <$20,000: 22% $20,000-49,999: 30% $50,000+: 48% Education <2-year college: 55% 2-year college+: 45% Distance from clinic <13 miles: 28% 13+ mi: 72%	NR
Unger et al.	2013	% group enrolled onto CCT: 65+: 5.4% Of evaluable respondents: 65+: 22% Of evaluable respondents: 65+: 22%	% group enrolled onto CCT: White/other: 9.0% AA: 11.1% Of evaluable respondents: W: 94.4% AA: 2.5% AAPI: 1.1% NA: 0.4% Other: 1.6% Of evaluable respondents: W: 94.4% AA: 2.5% AAPI: 1.1% NA: 0.4% Other: 1.6%	% group enrolled onto CCT: M: 5.6% F: 11.1% Of evaluable respondents: F: 62% Of evaluable respondents: F: 62%	NR	% group enrolled onto CCT: <$50K: 7.6% $50K+: 10.0% <college: 7.9% college+: 9.6% Of evaluable respondents: <$50K: 32% <2-year. college degree: 34% Of evaluable respondents: <$50K: 32% <2-year college degree: 34%	% group enrolled onto CCT: 0-1: 10.1% 2+: 7.5% Of evaluable respondents: 0-1: 59% 2+: 41% Of evaluable respondents: 0–1: 59% 2+: 41%
VanderWalde et al. (2022)	2022	CCT enrollees: Median: 60 %65+: 39%	NR	NR	NR	NR	Account for intersecting role of disease site and # trial modalities
Wagar et al.	2022	Of enrollees: M: 60	Enrollment fraction by group: NHW: 1.519% NHB: 0.473% Hispanic: 0.338% AAPI: 2.379%	AFAB	NR	NR	NR
Yonemori et al.	2010	Median (Japan trials) = 59 Median (US trials) = 55 Median (Japan pop) = 59 Proportion >65 in Japan accruals: 35% Proportion >65 in US accruals: 28%	Japan USA (otherwise NR)	NR	NR	Briefly report on effects of SES intersecting with older patient underrepresentation	Report on effects of physical and psychological comorbidity impairing older patients disproportionately
Yekedūz et al.	2021	NR	Of CCT enrollees: Black: 2.1% Asian/Other: 19.4% Of population: Black: 9.8% Asian/Other: 8.1%	Of CCT enrollees: F: 36.0% Of population: F: 49.6%	NR	NR	Of CCT enrollees: HBV: 1.3% HCV: 0.8% HIV: NR Brain metastases: 1.6% ECOG <2: 82%
Zafar et al.	2011	Median: 71	Caucasian: 87% AA: 12% Other: 1%	M: 63% F: 37%	NR	NR	PS 0: 13% 1: 59% 2: 16% 3: 11% 4: 1% Comorbidities CV: 66% Renal: 6% Hepatic: 1% Hematologic: 3% Endocrine: 30%
Zhao et al.	2024	Total DMA: −8.15	NR	NR	NR	Briefly mention intersecting role of financial strain	Address intersecting role of comorbidities and disease site
Zullig et al.	2016	Of accruals: *M* = 57.8	Of accruals: White: 2.37% enrollment out of new cases Minority: 2.21% enrollment out of new cases	Of accruals: M: 1.46% enrollment out of new cases F: 3.25% enrollment out of new cases	NR	Of accruals: Q1 (fewest uninsured): 2.22%% enrollment out of new cases Q2: 2.43% enrollment out of new cases Q3: 2.49% enrollment out of new cases Q4 (most uninsured): 2.16% enrollment out of new cases	NR
Zuniga et al.	2020	NR	Of accruals: W: 80% B: 17% Other: 4% Of incident cases: W: 80% B: 15% Other: 5%	Men	NR	NR Report briefly on intersection between race and access to resources	NR

*Note:* Abbreviations included in this table are utilized as follows, listed alphabetically: AA = African American; AAPI = Asian American or Pacific Islander; ACOSOG = American College of Surgeons Oncology Group; AFAB = assigned female at birth; AI/AN = American Indian/Alaska Native; AMAB = assigned male at birth; AML = acute myeloid leukemia; ALL = acute lymphocytic leukemia; API = Asian or Pacific Islander; B = Black; CCC = comprehensive cancer center; CCT = cancer clinical trial; COPD = chronic obstructive pulmonary disease; CV = cardiovascular; DLBCL = diffuse large B-cell lymphoma; ECOG = Eastern Cooperative Oncology Group; edu = education; F = female; IQR = interquartile range; M = male; NCI = National Cancer Institute; NH = non-Hispanic; NIH = National Institutes of Health; NR = not reported; O = Other; PCa = prostate cancer; PL = poverty line; PS = performance status; SES = socioeconomic status; SGM = sexual and/or gender minority; W = White; WTP = willingness to participate; y.o. = years old.

### Quality and bias assessment

The Mixed-Methods Appraisal Tool (MMAT) [29] was applied across studies to ensure uniform quality ratings across while affording flexibility appropriate to specific article type. The MMAT includes five sets of five-item criteria, with one set applied to each article reviewed contingent on its specific study design. Fulfillment of each of the five criteria for a given study design yields one point. As such, scores range from “0” to “5,” with higher ratings indicating stronger evidence quality. Case-control, cohort, cross-sectional, and meta-analytic studies were assessed using MMAT criteria directed toward nonintervention, descriptive analyses. While this iteration of the MMAT has not been applied to reviews specific to CCT representation, multiple versions of the MMAT have been utilized in recent reviews addressing cancer health disparities [30–32].

## Results

### Article selection

**Figure 1.**
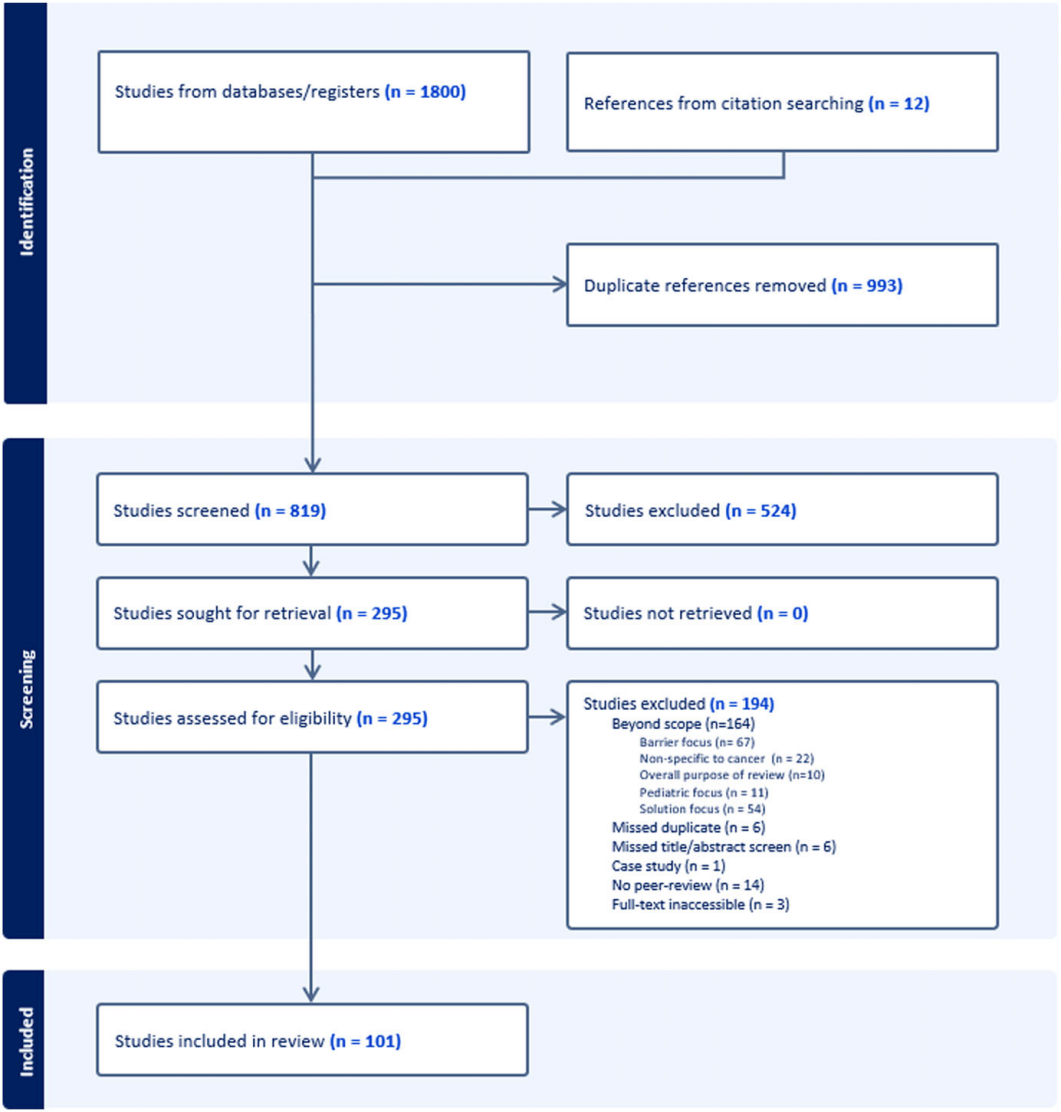
PRISMA flow diagram of selected articles.

The combined search strategies yielded an initial 1,812 articles. Nine hundred ninety-three duplicates were removed, including articles from the 2021 search identified in the 2024 search. Eight hundred nineteen titles and abstracts were reviewed for relevance. Two hundred ninety-five articles underwent full-text screening, with 194 studies excluded as detailed in the PRISMA flow diagram (Figure 1). A resulting total of 101 articles were included in the review.

### Study information

Approximately 66% of included studies (*n* = 67) primarily addressed tumor-directed, therapeutic trials, with 39.6% providing specific information on intervention types studied (*n* = 40). Only 12.8% of studies (*n* = 13) explicitly addressed trials with at least one supportive care, psychosocial, or behavioral component, with 5.0% of all studies (*n* = 5) exclusively focused on such CCTs. Approximately 18.8% of studies provided minimal detail on trial type eligibility criteria (*n* = 19). This information is summarized in Table 1.

Case-control studies – that is, studies examining differences between participants and non-participants using patient data repositories – constituted 44.6% (*n* = 45) of articles, with most utilizing population data to compare characteristics between CCT participants and corresponding oncologic populations. Nearly 29% of studies were conceptualized as cohort designs (*n* = 29, i.e., using patient data to evaluate predictors of CCT enrollment over time), and 7.5% (*n* = 7) were cross-sectional studies. Twenty-one studies were meta-analyses of aggregate demographic, socioeconomic, or medical characteristics across published trials (20.8%). Where classification according to these descriptions was ambiguous, our authors relied on self-identification of study design by the cited authors. This information is detailed in Table 2.

Approximately 81% (*n* = 82) of studies included race and/or ethnicity as a primary focus. In comparison, 54.4% (*n* = 55) addressed age, 35.6% (*n* = 36) addressed at least one socioeconomic indicator, 30.7% addressed sex or gender (*n* = 31), and 26.7% (*n* = 27) addressed at least one indicator of disability as CCT inequity targets. Only one eligible study addressed SGM status (0.99%) even following several modifications of advanced search strategies (Supplementary Table 1). The availability of social, economic, and medical characteristics across studies is detailed in Table 3.

### Quality assessment

Across all 101 studies, the quality mean MMAT score calculated was 4.59, with a median of 5. Score distribution was as follows: 5: 65.3% (*n* = 66), 4: 28.7% (*n* = 29), 3: 5.9% (*n* = 6), 2: 0.0% (*n* = 0), 1: 0.0% (*n* = 0). Quality ratings are summarized in Table 1.

### Synthesis of findings

### Race and ethnicity

Extant literature reflects robust evidence of CCT underrepresentation among patients of color, with mixed findings on representativeness across specific racial and ethnic minority groups. Early studies reflect lower enrollment among patients of color across multiple diagnostic sites, including in therapeutic lung, breast, colorectal, lymphoma, leukemia, and reproductive system CCTs [33], with some revealing decreased participation among patients of color across time (e.g., Baquet: 8.9% annual decrease among Black patients) [34]. Similar early trends are documented in surgical breast, colorectal, and thoracic CCTs [35]. Across the 50 largest National Cancer Institute (NCI) CCTs from 1996 to 2002, Black, Hispanic, and Asian American or Pacific Islander (AAPI) patients were all less likely to enroll in lung and colorectal CCTs, with Black and Hispanic women also less likely to enroll in breast trials [36]. These data indicated the poorest representation among Hispanic patients overall, and lower prostate CCT enrollment – a disparity not observed among other minority groups. This case-control study also showed a proportional decline in CCT enrollment among patients of color despite increased overall CCT participation from 1998 to 2002 [36]. While other evidence supports attenuation of some of these inequities with clinical cooperative group efforts (e.g., Newman: % Black CCT participants vs. cancer population: 10.5% and 9.4%) [37], early research consistently reflects national CCT underrepresentation among patients of color across various cancers.

Studies from the following decade demonstrate continued CCT underrepresentation among patients of color, adjusting for other relevant characteristics [38,39]. Longitudinal data emphasize stagnant therapeutic lung CCT enrollment among Black, Hispanic, and AAPI patients from 1990 to 2012, even with NCI cooperative group support [40]. Similar studies indicate worsening representation of Black women in gynecologic CCTs up to 2013, demonstrating 4.5–15 times lower enrollment than expected [41]. A meta-analysis from this period suggests still poorer trends, reporting 6.5 and 18.5 times lower enrollment than expected for Black women in cervical and ovarian trials, respectively, with representation worse from 2015 to 2018 compared to the late 1990s [4]. Other studies corroborate underrepresentation among patients of color in prostate, breast, colorectal, pancreatic gastric, hematologic, myelodysplastic, and varied sample CCTs at the catchment area level [42,43], in multi-site and multi-trial pharmacologic studies [44,45], Food and Drug Administration (FDA) CCTs with pharmaceutical sponsors [46], and in NIH CCTs from 1999 to 2019 [5]. Meta-analytic studies corroborate persistence of these inequities, reflecting poorest representation among Black and Hispanic patients in various therapeutic breast, colorectal, lung, prostate, pancreatic, renal, melanoma, and multiple myeloma CCTs, with such data collectively spanning 1981 to 2017 (e.g., Guerrero et al.: Not Reported, White, Black, and Hispanic CCT enrollment fractions [EF], respectively: 66.95%, 25.94%, 1.08%, 0.16%).[47,48]. Other national gynecologic CCT data not only accentuate Black and Hispanic underrepresentation but also larger disproportionate effects on Hispanic women with uterine and cervical cancers [49]. State-level studies reveal similar trends, with women of color less likely to enroll in early-stage breast radiotherapy CCTs overall, with Hispanic, then AAPI, then Black women, respectively, showing the lowest representation [50]. However, other findings during this period indicate the highest relative underrepresentation among AAPI, then Hispanic, then Black patients across breast, prostate, colorectal, and lung CCTs in national databases [6].

A few studies prior to 2021 suggest minimal inequities in CCT representation among patients of color with certain diagnoses, locations, and trial types. One national study reports no racial or ethnic differences in *opportunities* to participate in breast CCTs from 2013 to 2014 [51], with similar findings regarding prostate CCTs in earlier years [33,35]. A case-control analysis of FDA-approved therapies for breast, lung, colorectal, and prostate cancers showed persisting underrepresentation among patients of color relative to non-Hispanic White patients, though with recent improvements (% participants of color, 2008–2013: 20%; 2014–2017: 29%) [45]. Other evidence emphasizes representative accrual to surgical breast, thoracic, and sarcoma CCTs among Black and Hispanic patients [52], as is observed in NCI Community Cancer Centers Program CCTs overall [53]. Some findings during this period suggest equitable representation among patients of color in therapeutic lung CCTs, despite participation inequities in five other diagnostic sites [5]. Nonetheless, nonsurgical breast CCTs reliably demonstrate worsening representation among patients of color despite progress in other cancers (e.g., Zullig: 1996: < 1% vs. 2009: 3.5% enrollment difference between White and minority women, *p* < .001) [54]. Other studies reporting nonsignificant participation differences in some areas emphasize persisting *trends* toward underrepresentation among patients of color where typically observed [33].

Studies published within the past three years corroborate the intractability of CCT underrepresentation among patients of color while providing further nuance surrounding these inequities. State and national cohort, case-control, and meta-analytic studies of overall CCT representation evidence participation inequities that disproportionately impact Black [55–59] and/or Hispanic [47,48,56,57,59,60] patients in phase I [57], II [61], and III [59]; radiation [56,61]; drug [55,58,59]; brachytherapy [62]; and general CCTs [48,60] utilizing updated datasets and study repositories (e.g., Bero: Black proton participants vs. population 6.0% vs. 12.7%; Choradia: Hispanic participants vs. population: 7.1% vs. 13%; ). While some studies note *mild* representative improvement among Black [60,63] and Hispanic patients (e.g., Saphner: no significant inequities in White, Black, and Hispanic accrual: 90.4%, 6.6%, 1.9%; *p* = .078) [63], others demonstrate worsened representation in common cancers over time (e.g., 2009 vs. 2011–2015) [47]. Other case-control and meta-analyses emphasize underrepresentation among AAPI, Native Hawaiian, and American Indian/Alaska Native patients, in recent, CCTs for various prevalent cancers [48,56,64]. Still other findings evidence minimal underrepresentation among patients of color [63] and demonstrate even higher CCT participation among Asian patients, though such results have been primarily limited to singular institutions diagnostic sites, or trial types (e.g., Wagar: polymerase inhibitor CCT EF: White: 1.5%, Black: 0.47%, Hispanic: 0.33%, AAPI: 2.38%) [47,57,59,63].

Contemporary, cancer-specific studies reveal the importance of tumor site in dictating such inequities. Recent cohort, case-control, and meta-analytic studies of the most prevalent cancers continue to demonstrate underrepresentation among Black [62,65–72], Hispanic [62,65–70,72], Asian [62,72,73], and American Indian and Alaskan Native [62,67,69] individuals in breast [62,66–68,71,74], lung [65,67–71], and prostate CCTs (e.g., Ajewole: FDA oral chemotherapy CCT enrollment composition, 2009–2019: White: 71.5%, Black: 2.5%, Hispanic: 2.3%; Ladbury.: brachytherapy CCTs, enrollment incident disparity: Asian: −2.65%) [62,65,67,71,72,75–77]. Similar patterns are observed in understudied cancers, with CCT underrepresentation among Black [68,72,78–87], Hispanic [68,72,78–80,82,83,86], and Asian [72,80,86] patients with gastrointestinal [65,67,68,73,88], hepato-biliary [78,79], pancreatic [68,78,89,90], gynecologic [62,80,91], HPV-associated oropharyngeal [92], renal and urologic [72,87,93], hematologic [68,81–85], and neurologic [86] malignancies. Some evidence suggests potential mitigation of such inequities for certain cancers in recent years [65,80,86,94–97], particularly among Asian individuals (e.g., Javier-DesLoges: breast, colorectal, lung, and prostate participation odds ratios [OR], 2000–2004 vs. 2015–2019: Black: 2.19, 1.15, 1.54, 1.14; Hispanic: 3.32, 2.46, 2.21, 1.70; Asian: 1.94, 2.48, 3.88, 1.64) [47,65,67,85]. Nonetheless, such findings are primarily limited to studies with strong infrastructural support or smaller, singular institutional studies, while other contemporary studies reveal stagnation or worsened disparities over time (e.g., Owens-Walton: unchanging representation quotients from 2000 to 2017) [72,74].

Limited data reflect mixed findings regarding representation among patients of color in psychosocial CCTs. Some such evidence suggests minimal enrollment inequities between non-Hispanic and Hispanic women; however, even these data reflect higher attrition risk for Hispanic and immigrant women [98]. An institutional study of all cancers identified no racial/ethnic participation inequities among patients who were eligible for two pain and symptom-focused CCTs; however, patients of color were more likely to be *ineligible* [99]. Recent analyses suggest that psychosocial CCT representation among patients of color may be particularly contingent on intervention type, target population, and funding. For example, national evidence indicates Black underrepresentation in prostate exercise, advanced disease, and nongovernmental CCTs; adequate representation in dietary and multi-component trials; and disproportionately higher participation in pelvic floor muscle training, localized disease, and government-funded trials relative to their non-Black counterparts [100]. While observing poor representation among patients of color overall, a meta-analysis of integrated palliative CCTs suggests mitigated underrepresentation among Black patients compared to therapeutic CCTs (EF: 5.7% vs. 3.0%) [101], with similar, state-level results observed among Black men in behavioral CCTs [94]. Analyses of two psychosocial CCTs among women with gynecologic, gastrointestinal, and thoracic cancers demonstrate parallel trends, with even higher enrollment among Hispanic patients [102]. Nonetheless, other evidence investigating breast, lung, prostate, and colorectal CCTs reveals trends toward *poorer* representation among Black patients in supportive care trials compared to tumor-directed studies [6].

### Age

Strong evidence demonstrates CCT underrepresentation among older adults across time. Early such inequities are observed in NCI Cooperative Group trials in prevalent cancers, with patients 65 to 74 and patients older than 75 years old, respectively, exhibiting progressively lower accrual to nonsurgical, therapeutic trials compared to younger patients [36]. Such findings are replicated in later general CCT samples [13,34], surgical CCTs [35], and NCI, state-specific data further classifying older age subgroups [33,34]. Early large studies corroborate these trends across cancer types and within drug-specific trials, with underrepresentation among older adults relative to their incident populations[45,103–105] and lower likelihood of CCT enrollment with age [53], with progressively greater underrepresentation (e.g., Talarico: participants vs. population, respectively: 65+: 25% vs. 60%; 75+: 4% vs. 31%) [105].

Contemporary literature corroborates these findings, reflecting continued CCT underrepresentation among older adults over time. Recent institutional, state, and national cohort, case-control, and meta-analytic studies reveal persisting underrepresentation among older adults in surgical [39], drug [42,66,106], brachytherapy [62], and other trial types [65,94,51]; phase I [57], II [107], and III[108–110] trials; and multimodal [107] CCTs in general (e.g., Baldini: CCT referral vs. population 70 +: 17.7% vs. 50%) [42,57,58,63,106,107,111,112]. Such findings are replicated in specific cancers, including breast [42,65,66,39,108,113], gynecologic [49,51], lung [65,96,108], prostate [65,76,94,108], pancreatic [89,90], hepatic [79], gastroesophageal [114], gastrointestinal [42,65,73,108,115], renal [87], skin [109], head and neck [116], other solid organ [5,107], and hematologic cancers [5,110,117], with further evidence of greater inequities among the oldest groups [115].

Nonetheless, other recent studies report no age differences in CCT enrollment, especially controlling for relevant covariates (e.g., Dudipala: OR: 1.023) [44,50,92,97,118,119]. However, these findings have primarily been exclusive to one institution, diagnosis, or state. Further, evidence of more equitable age representation overall is qualified in persistent inequities relative to the incident population for that specific cancer[40], or among patients initially referred to [40], eligible for [102,113], or discussed for such trials [97,112]. Furthermore, other longitudinal and population-based studies demonstrate stagnated or worsened age inequities in CCT participation over time, particularly among the oldest patients (e.g., Zhao et al. median age difference [DMA] between participant and incident disease median age: −8.15; US DMA before 2017 vs. after 2017: −5.90, −8.00) [49,108,111].

### Socioeconomic status (SES)

Early national case-control analyses reflect breast CCT underrepresentation among low-SES patients by multiple indicators, including area poverty, unemployment, income, education, and individual government insurance [120]. Other site-specific studies document similar findings in various cancers, with lower CCT participation associated with higher material deprivation and lower social class (e.g., Mohd Noor.: Index of Multiple Deprivation [IMD] = 5 enrollment OR: 0.53, relative to least deprived IMD = 1) [34,119]. Another national, cross-sectional study revealed strongly prohibitive effects of low income on breast, colorectal, prostate, and lung CCT participation, controlling for other variables (< $50,000 income vs. $50,000+: OR: 0.73) [13], with progressively larger disparities among patients with the lowest incomes [13,118]. State analyses extend similar findings to area income in breast, genitourinary, gastrointestinal, and myelodysplastic CCT enrollment (e.g., Brierley et al.: average median income, participants vs. non-participants: $68,896 vs. $61,241) [43,121]. Other earlier studies reveal how unemployment, lower educational attainment [13,122], and governmental insurance [42,49] predict CCT underrepresentation in breast and other common cancers.

Contemporary studies within the past three years have increasingly focused on and further substantiated CCT underrepresentation among lower SES patients. Multilevel cohort, case-control, and meta-analytic studies evidence the effects of lower area income (e.g., Hue: stage IV participants vs. non-participants < $40,227: 11.4% vs. 19.1%) [63,87,89,112,123], education (e.g., Eskander: CCT participation, higher vs. lower high school attainment OR: 2.0) [73,78,80,87,89,90,94,112], insurance (e.g., Shinder: CCT participation, uninsured, Medicaid, or Medicare vs. private insurance ORs, respectively: 0.57, 0.43, 0.59) [70,73,78,87,90,96], or overall SES (e.g., Kwak: CCT participation, lowest [1] vs. median [4] SES group OR: 0.60) [63,70,82,96] on breast [66], prostate [77,94], lung,[70,96] gastrointestinal [73], pancreatic [78,89,90], hepatic [73,78], gynecologic [80], renal [87], brain [123], hematologic [83], and mixed CCT underrepresentation [57]. Other meta-analyses emphasize how limited SES reporting in CCTs significantly compromises research regarding its effects on representation [77,88].

Nonetheless, other studies present contrasting findings. data have shown higher breast CCT enrollment among Medicaid-eligible and lower-education patients [38]. Other studies have reflected higher surgical breast CCT participation with higher area education, but lower income [39], with similar income findings in gynecologic trials [80]. Some contemporary studies have observed no SES impact on CCT enrollment (e.g., Perni et al. participation OR, $100,000 median income vs. < $50,000: 1.28) [57] or attenuated effects in multivariate models [63,87,89], though these studies only examined socioeconomic factors as covariates. While the most equivocal evidence appears in the relationship between income and CCT participation, recent authors conceptualize such findings in reliance on area, rather than patient, indicators due to systemic data deficiencies [80]. Despite the nuances observed in these mixed findings, the literature provides growing evidence of socioeconomic CCT inequities by various indicators.

### Sex

Mixed literature on sex-related CCT inequities suggests contingency of representation on cancer and trial type. For example, early national data suggest higher therapeutic CCT enrollment among men with colorectal and lung cancers (participation, men vs. women OR: colorectal, lung, respectively: 1.30, 1.23) [36] with similar results replicated in center-specific analyses [124]. However, other early evidence regarding surgical CCTs reflects the reversal of this trend, with women five times more likely than men to enroll overall in a combined, national breast, colorectal, lung, and prostate sample [35]. Other data provide further insight into contrasting results, suggesting greater overall CCT participation among men, though lower enrollment compared to women when examining sex-specific cancers [34].

More recent studies have observed more equitable CCT representativeness across sexes. National cohort and meta-analytic studies addressing various cancers, including sex-specific [44] and rare diagnoses [117], reflect minimal sex differences in representation (e.g., Costa: observed-expected ratio, % male participants: 1.03). Similar evidence has emerged in psychosocial CCTs, revealing no sex differences in participation (e.g., Huang: % eligible enrolled in symptom CCT, within each sex: women: 75%, men: 78%) [99]. Longitudinal analyses reveal improvement in lung CCT representation over time among women younger than 65 years old (overall enrollment disparity difference between sexes reduced 0.07 to 0.03, 1994–2012) [40], as is consistent with equitable sex representation among younger patients in earlier lung and other CCTs [36]. Some state-specific evidence reflects even higher therapeutic lung, colorectal, and sex-specific CCT enrollment among women relative to men [54], as with the aforementioned surgical CCT findings [35]. Nonetheless, women’s underrepresentation persists in certain rarer cancers, such as myelodysplastic syndrome [43] or HPV-associated oropharyngeal CCTs (e.g., Gordis: % total female enrollees: 11.8%) [92]. Conversely, other data reflect disadvantages for men for certain CCT types across cancers, such as eligibility for chemoradiation trials [125] and participation in sex-related CCTs [34]. FDA approvals between 2008 and 2017 similarly demonstrate attenuated inequities when including sex-related CCTs, while simultaneously revealing worsened women’s representation over time when exclusively examining trials for cancers affecting all sexes (% women: 2008 to 2013: 47%, 2014 to 2017: 37%) [45].

Studies within the past three years continue to reveal minimal sex-related CCT inequities. Multiple institutional, state, and national cohort and case-control studies suggest equitable CCT participation across sexes in colorectal [68], lung [68–70], pancreatic [68,89], neurologic [123], hematologic [68,83,85], and mixed samples [63], with some analyses suggesting higher representation among women (e.g., Saphner: participation OR, men vs. women: 0.70) [63,70]. However, some of these findings are restricted to specific institutions, with their results challenged by more nationally representative analyses suggesting persistent underrepresentation among women in colorectal [65,88], lung [65], neurologic [86], and hematologic CCTs [84]. Additional studies document lower participation among women in hepatic [79], head and neck [95], and renal CCTs [87], in addition to women’s underrepresentation in overall therapeutic [60], radiation [56], phase II and III [57], non-sex- [63], and sex-specific diagnostic CCTs [60]. Though recent evidence of improved representation among women is qualified by these contrasting findings, contemporary results suggest *partial* mitigation of such inequities over time for certain diagnostic sites (e.g., Javier-DesLoges: women’s participation OR, 2015–2019 vs. 2000–2014: 1.38, with remaining inequities relative to men [OR: 0.89]) [65,86].

### Ability, staging, and functional status

Until the past three years, few studies had examined indicators of ability status as direct contributors to CCT participation, typically focusing on staging (i.e., measured by tumor size, lymph node presence, and/or metastases) [126], comorbidity, and more rarely, performance status ratings. Earlier findings evidence higher participation in breast [50,51,113,127], colorectal, lung, prostate [13], and multiple myeloma CCTs [117] among patients with lower staging or fewer comorbidities (e.g., Unger: participation OR, comorbidity score: 0.81), though primarily examine such indicators as covariates. Some institutional analyses characterize exclusionary comorbidities as restrictive to CCT participation across multiple cancers [122], while other data document positive relationships between symptom risk and therapeutic CCT enrollment in rarer cancers (e.g., Brierley: participation OR, high vs. very low risk: 1.88) [43]. Still, other investigators report no association between disease characteristics, comorbidities, and CCT participation [33], though these early studies still conceptualize such ability proxies as covariates, rather than key predictors.

While evidence remains scarce compared to other marginalizing indicators, contemporary studies have increasingly documented relationships among comorbidity, functional impairment, and CCT participation. National cohort, case-control, and meta-analytic studies reveal the potentially restrictive impact of comorbidity burden or associated lower performance status on pancreatic,[78,89,90], breast [66], lung [128], hepatic [78], gynecologic [80], other gastrointestinal and genitourinary [128], renal [87], head and neck [116], solid organ [129], and overall CCT representativeness (e.g., Green: % comorbidity score = 0, participants vs. non-participants: 69.2% vs. 51.6%) [58,112]. Other analyses, while not directly centering ability proxies as enrollment predictors, evidence the covarying impact of performance status on CCT participation (e.g., Bruno: lung participation OR, Eastern Cooperative Oncology Group [ECOG] score, 2 vs. 0: 0.27) [130]. Still other recent results evidence *positive* relationships between comorbidity burden and CCT participation, though these findings are exclusive to one state and disease site [95].

### Intersectionality in CCT participation inequities

The above-summarized data provide robust evidence of persistent CCT underrepresentation among patients of color and older adults, with mixed evidence of changing representativeness over time across diagnostic sites and trial types. Recent evidence reveals similar relationships between SES and CCT participation, demonstrating how lower education, inadequate insurance, and to a smaller extent, lower income, may further stifle CCT representativeness. While sex disparities have negatively impacted CCT participation depending upon cancer type, some contemporary studies evidence more equitable CCT representation in common cancers. While the singular impacts of such factors quantitatively vary, the interactivity among these social, economic, and medical marginalizing indicators further complexifies CCT representativeness.

This review characterizes the nexus among race, ethnicity, and SES as one of the most intricate intersections in determining CCT representation. Early breast CCTs have revealed diminishing underrepresentation among Black patients after considering area poverty, unemployment, and Medicaid coverage (participation OR, Black vs. White: 0.99) [120]. Later population data corroborate such findings, illustrating partial attenuation of Black and Hispanic underrepresentation in surgical breast CCTs when accounting for income and education [39], as well as insurance [131]. Institutional analyses of multiple cancers have demonstrated resolution in CCT underrepresentation among patients of color after accounting for age, sex, and deprivation index [119]. However, other evidence reveals underrepresentation among *higher* income and *privately* insured Black and Hispanic women compared to their less affluent counterparts in gynecologic [49,98] and breast CCTs (e.g., Fayanju: participation OR, Black and Hispanic, respectively, median income $63,000+ vs. < $38,000: 0.45, 0.19) [39]. These findings constitute a reversal of typically observed relationships, wherein racial, ethnic, and socioeconomic marginalization multiplicatively serve to restrict CCT participation with concurrent marginalization, rather than poorer participation among *higher* SES women of color. A meta-analysis of FDA approvals, regardless of SES, emphasizes the intersection among sex and minoritized identity, with the greatest underrepresentation observed among women of color in prevalent cancers (i.e., % Black participants breast sample: 2%) [45].

Studies within the last three years have increased explicit efforts to explore the interactive influences of racial, ethnic, and socioeconomic marginalizing indicators on CCT participation, while similarly indicating nuanced results across diagnostic sites. Multilevel cohort, case-control, and meta-analytic studies demonstrate the simultaneous impact of minoritized race/ethnicity, lower area SES, and inadequate insurance coverage in limiting breast [66], gynecologic [80], pancreatic [90], and renal [87] CCT participation (e.g., Khadraoui: participation ORs, racial/ethnic minority vs. White: Black: 0.70, Hispanic: 0.53, Asian: 0.44, Other: 0.48; education, 15.3%+ vs. < 5.0% without high school education: 0.41). Similar studies demonstrate partial contingency of hematologic CCT underrepresentation among people of color on lower area income or insurance coverage [82]. Still, other recent studies corroborate persistent CCT inequities that disproportionately affect among women of color regardless of income, in gastrointestinal trials [73], as is consistent with earlier breast and gynecologic CCTs [49,39].

Studies investigating relationships among race, ethnicity, and SES in determining CCT representation have increasingly revealed potential contributions of disease characteristics, comorbidity burden, and performance status. For instance, early analyses demonstrate how controlling for advanced disease diminishes otherwise observed racial CCT inequities [37,50]. Similar interactive relationships have been observed in early case-control studies regarding lung CCTs, interpreting underrepresentation among Black and other patients of color within the intersections among race, SES, insurance, comorbidity, and performance status [124]. Other national data corroborate higher comorbidity among Black patients considered for CCTs (medical comorbidity presence, OR: 1.53) [53]. More recent analyses directly explore how ability indicators color the intersectional effects of race, ethnicity, and SES on CCT representation [66,80,87,94,129]. While some such studies reveal how higher staging and comorbidity may further limit CCT participation among minoritized or lower SES patients (e.g., Yekeduz: % Black participants vs. population: 2.1% vs. 9.8%, with 82% total sample with ECOG 0–1) [66,72,80,87,129], others offer opposing evidence among certain underserved populations. Specifically, some studies indicate increased CCT participation among patients of color with higher comorbidity burden and staging, such as Hispanic men with prostate cancer [94]. Still others indirectly examine complex, intersectional influences of comorbidity, illness characteristics, and ability on CCT representativeness, suggesting poorer overall CCT participation due to the COVID-19 pandemic, though with unexpected impacts on participation inequities (e.g., Choradia et al.: participation ORs, 2005–2020, each vs. White patients: Hispanic: 0.52, American Indian: 0.41, AAPI: 0.81; peak participation among these underserved in 2020, despite lowest year of enrollment across population) [60,68].

Such patterns are further influenced by age and sex, especially among older adults of color with an increased comorbidity burden. Early analyses demonstrate how older age compromises breast, colorectal, thoracic, and prostate CCT participation across racial and ethnic groups, though drives underrepresentation otherwise unobserved in younger patients among women of color [36]. Other investigators demonstrate how older age heightens gynecologic CCT attrition risk for Hispanic, but not for non-Hispanic, women [98]. Recent national cohort, case-control, and meta-analytic studies strengthen evidence of simultaneous underrepresentation regarding older age, comorbidity, performance status, and other marginalizing factors underpinning CCT underrepresentation (e.g., Kaanders: % participants with World Health Organization [WHO] 0–1 or Karnofsky performance score 90–100: 70%; median age, participant vs. population: 57, 64 [58,107,109,112,116], with some evidence emphasizing how *trial* characteristics themselves may limit participation among older adults with higher disease burden [107].

Regarding intersecting sex influences, some early state studies indicate elevated racial disparities among men relative to women in therapeutic trials for common cancers [34,54], with recent studies similarly accentuating how cancer sex-specificity may underpin racial and ethnic representativeness in radiation CCTs (i.e., Black underrepresentation observed in all CCT types *except* sex-specific female [13.1% sample] and male [18.4% sample] US trials) [56]. While quantitatively unexamined to date, contemporary studies have *begun* to comment on how relationships among these marginalizing factors may be furthermore impacted by sexual minoritization, through its influence on preexisting health and CCT eligibility [66]. Overall, relationships among social, economic, and medical marginalizing indicators in underpinning CCT inequities have gained increasing attention in recent years, with more investigators explicitly exploring the structural, intersectional context of such factors when interpreting their findings regarding CCT representativeness [72,39].

## Discussion

This review sought to describe CCT participation inequities via multiple modes of social, economic, and medical marginalization, including race, ethnicity, age, sex, SGM identity, SES, and ability. Its findings contribute novel insights regarding the impact of such factors on CCT inequities, including strengthened evidence for national CCT underrepresentation among racial and ethnic minority groups and older adults across various cancers and trial types. To a lesser, albeit increasing extent, these results reveal compromised CCT participation among lower SES patients across various metrics, especially education and insurance; however, these findings are dependent on aggregate, rather than individual, SES indicators. This review further offers insights into the effects of ability status on CCT participation, with a growing focus on comorbidity burden in recent years.

These findings reflect minimal to modest evidence of improvement in representativeness across the past several decades. While exhibiting some progress in racial, ethnic, and sex representativeness in certain intervention types, CCT inequities are observed across most cancers and study designs in recent large-scale analyses. Studies focused on CCT representation among the underserved have more than doubled within the past three years, while accentuating a persisting absence of data investigating such inequities among SGM patients. Nonetheless, while bolstering evidence of intractable CCT inequities across various other marginalizing indicators and cancers, contemporary investigations have increasingly provided more nuanced insights into their complex interplay in determining CCT representativeness.

More important than enduring inequities observed in a singular examination of each marginalizing indicator, however, is the intersection among these social, economic, and medical characteristics and their effects on CCT inequities. These results demonstrate the partial underpinning of CCT underrepresentation among patients of color by parallel preexisting socioeconomic and health disparities. Further, the literature illustrates how the intersection among racial/ethnic minority status, SES, and other marginalizing indicators may interactively predispose individuals with cancer to more aggressive disease, higher comorbidity, or poorer performance status, thus compromising CCT participation among the underserved. These conditions are further influenced by the strong relationship between age and higher ineligibility risk due to similar preexisting health inequities, and their heightened impact on CCT representation in their intersectional context with other marginalizing indicators. Despite an increased focus on these relationships in recent CCT literature, few articles explicitly allude to their intersectional, structural nature, with most studies addressing multiple marginalizing indicators as potential confounding covariates at best.

This review is the first to conceptualize existing CCT inequities across several modes of social, economic, and medical marginalization through an intersectional perspective. These findings accentuate how numerous marginalizing indicators limit CCT representativeness with multiplicative implications, further preventing equitable participation among those with overlapping experiences of social, economic, and medical oppression. Further, this review is uniquely underpinned by a central recognition of social inequality, context, power, and justice using intersectionality as a theoretical scaffold for understanding public health [25,26].

## Limitations

This review is limited in its absence of articles addressing CCT participation among SGM individuals, yielding only one study that transiently mentioned SGM identity as one factor affecting CCT participation while interpreting its results. While this may indicate limitations in the search strategies applied to this review, this absence of SGM studies persisted with extensive adjustments, thus likely reflecting large deficits in the literature itself. Another limitation is a lack of explicit investigation regarding the impacts of rurality on CCT participation – a crescent area of research important to understanding CCT representation through an intersectional perspective.

Other limitations consist in a low number of articles that specifically address (1) supportive care, psychosocial, behavioral, or quality of life interventions and (2) longitudinal retention in studies. Further, few included articles directly investigate relationships between social, economic, and medical marginalization through an explicitly intersectional perspective, primarily examining such interactive influences through reductive, additive models that merely control for covarying factors. These results are also qualified by the limitations in article quality evaluation. While the use of the MMAT for quality assessment accommodated the diversity of articles included, this flexibility inversely limits the standardization of ratings across various article types. Further, while intersectionality constitutes a necessary lens through which investigators must accurately view health inequities, optimal practices for quantification of such outcomes through this theoretical paradigm remain tenuous.

## Implications and future directions

This review characterizes the current state of the literature quantifying CCT participation inequities that disproportionately impact the underserved in cancer care. Its description of such inequities reveals little ambiguity in CCT underrepresentation among certain marginalized groups, especially among older adults, racial/ethnic minorities, and by some indicators, patients originating in lower SES areas or with greater disability. This review thus constitutes a strong foundation to further investigate underpinning barriers that sustain these inequities and potential solutions to dismantle them. Its findings accentuate the necessity of future research focused on (1) mixed evidence regarding specific social, economic, and medical indicators in determining CCT participation, (2) the role of intersectionality and underlying mechanisms in explaining such inequities, and (3) persistently understudied marginalized populations in the investigation of CCT representation, especially patients who are SGMs, of lower SES or rural origin, or live with comorbid disabilities. Additional research is necessary to understand the generalizability of such findings to CCTs beyond those that are tumor-directed and longitudinal participation patterns.

This review accentuates the persistence of CCT participation inequities across various vectors of social, economic, and medical marginalization through an intersectional perspective across the past four decades. As such, these findings emphasize the urgency of identifying and dismantling barriers that sustain these inequities. Through such efforts, investigators and clinicians may strive toward the eradication of inequities in cancer outcomes and equitable benefits from advancements in cancer care among the underserved.

## Supporting information

Hanvey et al. supplementary material Hanvey et al. supplementary material

## Supplementary material

The supplementary material for this article can be found at https://doi.org/10.1017/cts.2024.677.
