# Supplementary material for: The role of social, economic, and medical marginalization in cancer clinical trial participation inequities: A systematic review
Source: J Clin Transl Sci. 2024 Dec 20;9(1):e25. doi: 10.1017/cts.2024.677 (PMC11883616; doi:10.1017/cts.2024.677)
Supplement: Hanvey et al. supplementary material [file S2059866124006770sup001.docx]

**Supplementary Material**

**Supplementary Table 1.** Search Strategy by Database

| **PubMed** | (Neoplasms[MH] OR cancer*[tiab] OR neoplas*[tiab] OR malignan*[tiab] OR sarcoma*[tiab] OR carcinoma*[tiab] OR adenocarcinoma*[tiab] OR melanoma*[tiab] OR leukemia*[tiab] OR hamartoma*[tiab] OR tumor* OR lymphoma*[tiab] OR myeloma*[tiab] OR oncolog*[tiab] OR tumor[tiab]) AND (Clinical Trials as Topic[MH] OR "clinical trial"[tiab] OR "RCT"[tiab] OR "controlled trial"[tiab] OR randomiz*[tiab]) AND (Healthcare Disparities[MH] OR disparit*[tiab] OR inequalit*[tiab] OR inequit*[tiab] OR unequal*[tiab] OR imbalanc*[tiab] OR uneven*[tiab]) AND (Patient Participation Rates[MH] OR recruit*[tiab] OR accru*[tiab] OR enroll*[tiab] OR retention[tiab] OR retain[tiab]) AND (Minority Health[MH] OR Disabled Persons[MH] OR Emigrants and Immigrants[MH] OR Homeless Person[MH] OR Medically Uninsured[MH] OR African Americans[MH] OR Asian Americans[MH] OR Hispanic Americans[MH] OR Indigenous Peoples[MH] OR Sexual and Gender Minorities[MH] OR Vulnerable Populations[MH] OR Working Poor[MH] race[tiab] OR races[tiab] OR racial[tiab] OR ethnic*[tiab] OR "sexual minorit*"[tiab] OR "gender minorit*"[tiab] OR "LGBT"[tiab] OR gay*[tiab] OR lesbian*[tiab] OR bisexual*[tiab] or transgender*[tiab] OR age[tiab] OR "older adult*"[tiab] OR "younger adult*"[tiab] OR "socioeconomic status"[tiab] OR "SES"[tiab] OR income[tiab] OR education[tiab] OR occupation[tiab] OR employ*[tiab] OR disab*[tiab] OR "able-bodied"[tiab] OR "differently abled"[tiab] OR handicap*[tiab] OR "physical challeng*"[tiab] OR "physically challenged"[tiab] OR "intellectually challenged"[tiab] OR "intellectual challeng*"[tiab] OR "emotional challeng*"[tiab]) |
| --- | --- |
| **PsycINFO** | (**MeSH**: Neoplasms *OR* **abstract**: cancer* *OR* **abstract**: neoplas* *OR* **abstract**: malignan* *OR* **abstract**: sarcoma* *OR* **abstract**: carcinoma* *OR* **abstract**: adenocarcinoma* *OR* **abstract**: melanoma* *OR* **abstract**: leukemia* *OR* **abstract**: hamartoma* *OR* **abstract**: tumor* *OR* **abstract**: lymphoma* *OR* **abstract**: myeloma* *OR* **abstract**: oncolog* *OR* **abstract**: tumor) *AND* (**MeSH**: Clinical Trials as Topic *OR* **abstract**: "clinical trial" *OR* **abstract**: "RCT" *OR* **abstract**: "controlled trial" *OR* **abstract**: randomiz*) *AND* (**MeSH**: Healthcare Disparities *OR* **abstract**: disparit* *OR* **abstract**: inequalit* *OR* **abstract**: inequit* *OR* **abstract**: unequal* *OR* **abstract**: imbalanc* *OR* **abstract**: uneven*) *AND* (**MeSH**: Patient Participation Rates *OR* **abstract**: recruit* *OR* **abstract**: accru* *OR* **abstract**: enroll* *OR* **abstract**: retention *OR* **abstract**: retain) *AND* (**MeSH**: Minority Health *OR* **MeSH**: Disabled Persons *OR* **MeSH**: Emigrants *AND* **MeSH**: Immigrants *OR* **MeSH**: Homeless Person *OR* **MeSH**: Medically Uninsured *OR* **MeSH**: African Americans *OR* **MeSH**: Asian Americans *OR* **MeSH**: Hispanic Americans *OR* **MeSH**: Indigenous Peoples *OR* **MeSH**: Sexual and Gender Minorities *OR* **MeSH**: Vulnerable Populations *OR* **MeSH**: Working Poor *OR* **abstract**: race *OR* **abstract**: races *OR* **abstract**: racial *OR* **abstract**: ethnic* *OR* **abstract**: "sexual minorit*" *OR* **abstract**: "gender minorit*" *OR* **abstract**: "LGBT" *OR* **abstract:** gay* *OR* **abstract:** lesbian* *OR* **abstract:** bisexual* *OR* **abstract:** transgender* *OR* **abstract**: age *OR* **abstract**: "older adult*" *OR* **abstract**: "younger adult*" *OR* **abstract**: "socioeconomic status" *OR* **abstract**: "SES" *OR* **abstract**: income *OR* **abstract**: education *OR* **abstract**: occupation *OR* **abstract**: employ* *OR* **abstract**: disab* *OR* **abstract**: "able-bodied" *OR* **abstract**: "differently abled" *OR* **abstract**: handicap* *OR* **abstract**: "physical challeng*" *OR* **abstract**: "physically challenged" *OR* **abstract**: "intellectually challenged" *OR* **abstract**: "intellectual challeng*" *OR* **abstract**: "emotional challeng*") |
| **Web of Science** | (TI=(cancer OR neoplas* OR malignan* OR sarcoma* OR carcinoma* OR  adenocarcinoma* OR melanoma* OR leukemia* OR hamartoma* OR tumor* OR lymphoma* OR myeloma* OR oncolog* OR tumor) OR AB=(Neoplasms OR  cancer OR neoplas* OR malignan* OR  sarcoma* OR carcinoma* OR  adenocarcinoma* OR melanoma* OR leukemia* OR hamartoma* OR tumor* OR lymphoma* OR myeloma* OR oncolog* OR tumor)) AND (TI=("clinical trial" OR "RCT" OR "controlled trial" OR randomiz*) OR AB=("clinical trial" OR "RCT" OR "controlled trial" OR randomiz*)) AND (TI=(“health disparities” OR disparit* OR inequalit* OR inequit* OR unequal* OR imbalanc* OR uneven*) OR AB=(“health disparities” OR disparit* OR inequalit* OR inequit* OR unequal* OR imbalanc* OR uneven*)) AND (TI=(“patient participation rates” OR recruit* OR accru* OR enroll* OR retention OR retain) OR AB=(“patient participation rates” OR recruit* OR accru* OR enroll* OR retention OR retain)) AND (TI=(“minority health” OR “disabled persons” OR emigrants OR immigrants OR “homeless person” OR “medically uninsured” OR “African Americans” OR “Asian Americans” OR “Hispanic Americans” OR “Indigenous Peoples” OR “sexual and gender minorities” OR “vulnerable populations” OR “working poor” OR race OR races OR racial OR ethnic* OR "sexual minorit*" OR "gender minorit*" OR "LGBT" OR gay* OR lesbian* OR bisexual* OR transgender* OR age OR "older adult*" OR "younger adult*" OR "socioeconomic status" OR "SES" OR income OR  education OR occupation OR employ* OR disab* OR "able-bodied" OR "differently abled" OR handicap* OR "physical challeng*" OR "physically challenged" OR "intellectually challenged" OR "intellectual challeng*" OR "emotional challeng*") OR AB=(“minority health” OR “disabled persons” OR emigrants OR immigrants OR “homeless person” OR “medically uninsured” OR “African Americans” OR “Asian Americans” OR “Hispanic Americans” OR “Indigenous Peoples” OR “sexual and gender minorities” OR “vulnerable populations” OR “working poor” OR race OR races OR racial OR ethnic* OR "sexual minorit*" OR "gender minorit*" OR "LGBT" OR age OR "older adult*" OR "younger adult*" OR "socioeconomic status" OR "SES" OR income OR  education OR occupation OR employ* OR disab* OR "able-bodied" OR "differently abled" OR handicap* OR "physical challeng*" OR "physically challenged" OR "intellectually challenged" OR "intellectual challeng*" OR "emotional challeng*")) |
| **CINAHL** | (MH Neoplasms OR AB cancer OR AB neoplas* OR AB malignan* OR AB sarcoma* OR AB carcinoma* OR AB adenocarcinoma* OR AB melanoma* OR AB leukemia* OR AB hamartoma* OR AB tumor* OR AB lymphoma* OR AB myeloma* OR AB oncolog* OR AB tumor) AND (MH Clinical Trials as Topic OR AB "clinical trial" OR AB "RCT" OR AB "controlled trial" OR AB randomiz*) AND MH (Healthcare Disparities OR AB disparit* OR AB inequalit* OR AB inequit* OR AB unequal* OR AB imbalanc* OR AB uneven* OR AB represent* OR AB underrepresent*) AND (MH Patient Participation Rates OR AB recruit* OR AB accru* OR AB enroll* OR AB retention OR AB retain) AND (MH Minority Health OR MH Disabled Persons OR MH Emigrants OR MH Immigrants OR MH Homeless Person OR MH Medically Uninsured OR MH African Americans OR MH Asian Americans OR MH Hispanic Americans OR MH Indigenous Peoples OR MH Sexual and Gender Minorities OR MH Vulnerable Populations OR MH Working Poor OR AB race OR AB races OR AB racial OR AB ethnic* OR AB "sexual minorit*" OR AB "gender minorit*" OR AB "LGBT" OR AB gay* OR AB lesbian* OR AB bisexual* OR AB transgender* OR AB age OR AB "older adult*" OR AB "younger adult*" OR "socioeconomic status" OR AB "SES" OR AB income OR AB education OR AB occupation OR AB employ* OR AB disab* OR AB "able-bodied" OR AB "differently abled" OR AB handicap* OR AB "physical challeng*" OR AB "physically challenged" OR AB "intellectually challenged" OR AB "intellectual challeng*" OR AB "emotional challeng*") |
